# Supplementary material for: mTORC2–NDRG1–CDC42 axis couples fasting to mitochondrial fission
Source: Nat Cell Biol. 2023 Jun 29;25(7):989–1003. doi: 10.1038/s41556-023-01163-3 (PMC10344787; doi:10.1038/s41556-023-01163-3)
Supplement: Supplementary file 1 — Reporting Summary [file 41556_2023_1163_MOESM1_ESM.pdf]

nature portfolio

Corresponding author(s): RAJAT SINGH

Last updated by author(s): Apr 20, 2023

Reporting Summary

Nature Portfolio wishes to improve the reproducibility of the work that we publish. This form provides structure for consistency and transparency in reporting. For further information on Nature Portfolio policies, see our Editorial Policies and the Editorial Policy Checklist.

Statistics

For all statistical analyses, confirm that the following items are present in the figure legend, table legend, main text, or Methods section.

n/a	Confirmed	
☐	☒	The exact sample size (*n*) for each experimental group/condition, given as a discrete number and unit of measurement
☐	☒	A statement on whether measurements were taken from distinct samples or whether the same sample was measured repeatedly
☐	☒	The statistical test(s) used AND whether they are one- or two-sided<br>*Only common tests should be described solely by name; describe more complex techniques in the Methods section.*
☐	☒	A description of all covariates tested
☐	☒	A description of any assumptions or corrections, such as tests of normality and adjustment for multiple comparisons
☐	☒	A full description of the statistical parameters including central tendency (e.g. means) or other basic estimates (e.g. regression coefficient) AND variation (e.g. standard deviation) or associated estimates of uncertainty (e.g. confidence intervals)
☐	☒	For null hypothesis testing, the test statistic (e.g. *F*, *t*, *r*) with confidence intervals, effect sizes, degrees of freedom and *P* value noted<br>*Give P values as exact values whenever suitable.*
☒	☐	For Bayesian analysis, information on the choice of priors and Markov chain Monte Carlo settings
☒	☐	For hierarchical and complex designs, identification of the appropriate level for tests and full reporting of outcomes
☒	☐	Estimates of effect sizes (e.g. Cohen's *d*, Pearson's *r*), indicating how they were calculated

Our web collection on statistics for biologists contains articles on many of the points above.

Software and code

Policy information about availability of computer code

Data collection	Commercial acquisition softwares accompanying the following devices were used to collect data. Acquisition softwares for (a) Zeiss Axiolab 5 microscope with Axiocam 305 color camera for immunohistochemistry (Zeiss ZEN v3.7), (b) Leica TCS SP8 Confocal Laser Scanning Microscope for fluorescence images and live-cell videos (LAS X v.5.7.23225), (c) Zeiss Supra 40 Field Emission Scanning Electron Microscope to acquire transmission electron microscope images and videos (Zeiss SmartSEM v6.0) and 3D Modeling (3DMOD v4.9.10), (d) XF96 and X24 Seahorse analyzers (Agilent Technologies) to collect oxygen consumption rates (WAVE Pro v10.0.1.84; v2.6.1.56, respectively), and normalized to cell number determined by using Synergy HTX (BioTek) multi-mode plate reader (Gen5 v3.12), (e) StepOnePlus™ Real-Time PCR System (Applied Biosystems) for mRNA expression (StepOne v2.3).
Data analysis	Microsoft Excel v16.48, Microsoft Word v16.48, Microsoft PowerPoint v16.47 were used for processing and representation. Prism v8.4.3. was used for data analyses. Endnote X9.3.3. was used for assimilating citations. ImageJ v2.0.0-rc-69/1.52p was used for the quantification of western blot images. MitoTracker™, Red CMXRos fluorescence intensity, and mitochondria and/or ER shape parameters. The enrichment map was generated in Cytoscape v3.8.1 using Enrichment map plugin v3.3.0. Data handling and statistical analyses were performed using Python v.3.7.4 and scientific python stack: SciPy v.1.3.1, NumPy v.1.17.2, and Matplotlib v.3.1.1. Phosphosites showing significant regulation between groups were used to predict the kinase responsible for their catalysis using the iGPS software.

For manuscripts utilizing custom algorithms or software that are central to the research but not yet described in published literature, software must be made available to editors and reviewers. We strongly encourage code deposition in a community repository (e.g. GitHub). See the Nature Portfolio guidelines for submitting code & software for further information.

Data

Policy information about availability of data

All manuscripts must include a data availability statement. This statement should provide the following information, where applicable:

- Accession codes, unique identifiers, or web links for publicly available datasets
- A description of any restrictions on data availability
- For clinical datasets or third party data, please ensure that the statement adheres to our policy

The mass spectrometry proteomics data have been deposited to the ProteomeXchange Consortium via the PRIDE88 partner repository, and data are available via ProteomeXchange with identifier PXD041696. All the Python codes used in proteomic analyses are fully available on Github (https://github.com/MathieuBo/mTorc2_mito_fission). No restrictions.

Human research participants

Policy information about studies involving human research participants and Sex and Gender in Research.

Reporting on sex and gender	N/A
Population characteristics	N/A
Recruitment	N/A
Ethics oversight	N/A

Note that full information on the approval of the study protocol must also be provided in the manuscript.

Field-specific reporting

Please select the one below that is the best fit for your research. If you are not sure, read the appropriate sections before making your selection.

☒ Life sciences ☐ Behavioural & social sciences ☐ Ecological, evolutionary & environmental sciences

For a reference copy of the document with all sections, see nature.com/documents/nr-reporting-summary-flat.pdf

Life sciences study design

All studies must disclose on these points even when the disclosure is negative.

Sample size	A minimum of three independent experiments were performed unless otherwise stated. N numbers indicate biological replicates. Sample sizes were determined based on power calculations from previous studies in our laboratory. Measurements were taken from distinct samples except from live cell imaging experiments when the same cell was recorded for a period of time.
Data exclusions	Statistically-validated outliers were determined (via Prism v8.4.3) by Grubb's method (alpha=0.05) and those verified were eliminated.
Replication	All replication attempts in this study were successful. In vivo experiments comprising Con and RictorKO mice were performed 6 independent times including a minimum of n=3 mice per group per repetition. Experiments comprising mice expressing WT or Ser366Ala mutant NDRG1 were performed 3 independent times including a minimum of n=3 mice per group per repetition. Experiments comprising corn oil gavage were performed 6 times including a minimum of n=3 mice per group per repetition. Experiments comprising Bodipy FL C16-gavage were performed 3 independent times including a minimum of n=3 mice per group per repetition.
Randomization	All mice used in this study were randomly assigned to the different experimental groups. Equal sex and age were maintained across the different animal groups within each of the experiments.
Blinding	The persons performing the electron microscopy imaging were unaware of the sample identity. For experiments comprising confocal imaging acquisition and image/video processing, experimental settings (i.e., laser intensity, channel intensity, etc.) were standardized using the control cells as a reference, and similar settings were applied to the rest of the experimental groups/conditions within the experiment. Quantification for mitochondrial number and shape descriptors was performed blindly. For the rest of the analyses, blinding was not relevant because sample identification was required to conduct the analyses, e.g., for MAM preparations which require pooling to mice and western blotting.

Reporting for specific materials, systems and methods

We require information from authors about some types of materials, experimental systems and methods used in many studies. Here, indicate whether each material, system or method listed is relevant to your study. If you are not sure if a list item applies to your research, read the appropriate section before selecting a response.

Materials & experimental systems

n/a	Involved in the study
☐	☒ Antibodies
☐	☒ Eukaryotic cell lines
☒	☐ Palaeontology and archaeology
☐	☒ Animals and other organisms
☒	☐ Clinical data
☒	☐ Dual use research of concern

Methods

n/a	Involved in the study
☒	☐ ChIP-seq
☒	☐ Flow cytometry
☒	☐ MRI-based neuroimaging

Antibodies

Antibodies used

All primary and secondary antibodies were commercially available from the following sources: Rabbit polyclonal anti-AKT (#9272; Lot #30), Rabbit polyclonal anti-AKT1 (#2938; Lot #4), Rabbit polyclonal anti-AKT2 (#30638), Rabbit monoclonal anti-Phospho AKT (Thr308) (#13038; Lot #9), Rabbit monoclonal anti-Phospho AKT (Ser473) (# 4060; Lot #27), Rabbit polyclonal anti-AMPK? (#2532; Lot #21), Rabbit polyclonal anti-Phospho AMPK? (Thr172) (#2531; Lot #19), Rabbit polyclonal anti-p190-A RhoGAP (ARHGAP35) (#2513; Lot #1), Rabbit monoclonal anti-CDC42 (#2466; Lot #6), Rabbit monoclonal anti-CHOP (#5554; Lot #5), Rabbit monoclonal anti-CYTOCHROME c (#11940; Lot #4), Rabbit polyclonal anti-DYNAMIN I/II (# 2342; Lot #1), Rabbit monoclonal anti-DRP1 (#8570; Lot #5), Rabbit monoclonal anti-Phospho-DRP1 (Ser616) (# 4494; Lot #4), Rabbit monoclonal anti-Phospho-DRP1 (Ser637) (#6319; Lot #2), Rabbit monoclonal anti-DYKDDDDK Tag (#14793; Lot #5), Rabbit polyclonal anti-Phospho-eIF2α (Ser51) (#9721; Lot #21), Rabbit polyclonal anti-K48-linkage Specific Polyubiquitin (#4289; Lot #2), Rabbit monoclonal anti-MFF (#84580; Lot #1), Rabbit monoclonal anti-MFN2 (#9482; Lot #4), Rabbit polyclonal Phospho-NDRG1 (Thr346) (#5482; Lot #5), Rabbit monoclonal anti-OPA1 (#80471; Lot #1), Mouse monoclonal anti-PARKIN (#4211; Lot #8), Rabbit monoclonal anti-PKA C-α (#5842; Lot #4), Rabbit monoclonal anti-Phospho-PKA C (Thr197) (#5661, Lot # 3), Rabbit polyclonal anti-PKCα (#2056; Lot #5), Rabbit monoclonal anti-PKCδ (#9616; Lot #3), Rabbit monoclonal anti-PKCζ (#9368; Lot #4), Rabbit polyclonal anti-Phospho-PKCα/β II (Thr638/641) (#9375; Lot #5), Rabbit polyclonal anti-PKCδ (Thr505) (#9374; Lot #7), Rabbit polyclonal anti-PKCδ/θ (Ser643/676) (#9376; Lot #6), Rabbit polyclonal anti-Phospho-PKCζ/λ (Thr410/403) (#9378; Lot #10), Rabbit polyclonal anti-p70 (#9202; Lot #21), Rabbit monoclonal anti-Phospho-p70 S6 Kinase (Thr389) (#9234; Lot #12), Rabbit monoclonal anti-RAPTOR (#2280; Lot #11), Rabbit monoclonal anti-RHOA (#2117; Lot #6), Rabbit monoclonal anti-RICTOR (#2114; Lot #7), Rabbit monoclonal anti-S6 (#2217; Lot #10), Rabbit monoclonal anti-Phospho-S6 Ribosomal Protein (Ser235/236) (#4858; Lot #21), Rabbit polyclonal anti-?-Tubulin (#2144; Lot #7) and Rabbit monoclonal anti-TSC1 (#6935; Lot #4) were purchased from Cell Signaling Technology. Rabbit polyclonal anti-FACL4 (#ab227256; Lot #1035983-1), Rabbit monoclonal anti-CALRETICULIN (#ab92516; Lot # GR3287998-1), Rabbit polyclonal Anti-MTP18/MTFP1 (#ab198217; Lot # GR3381113-12), Rabbit polyclonal anti-VDAC1 (# ab15895; Lot # GR3452674-3), and Total OXPHOS Rodent WB Antibody Cocktail (#ab110413; Lot # 2101014434) were purchased from Abcam. Mouse monoclonal anti-eIF2α (#133132) was purchased from Santa Cruz Biotechnology. Mouse monoclonal anti-BiP/GRP78 (#610978; Lot #1277761) was purchased from BD Biosciences. Mouse monoclonal anti-XBP-1s (#647502; Lot #B131918) was purchased from Biolegend. Rabbit polyclonal anti-MFN1 (#GTX64398; Lot # 822103891) was purchased from GeneTex. Rabbit monoclonal anti-CDC42EP1 (#MA5-37968; Lot # XA3483164) and Rat monoclonal anti-mCherry (#M11217; Lot # XJ359389) were purchased from Invitrogen. Rabbit polyclonal anti-ARHGDIA (#MBS9413899; Lot #4801) was purchased from MyBioSource. Goat polyclonal anti-GFP (#NB100-1770; Lot #48180) was purchased from Novus Biologicals. Rabbit polyclonal anti-NDRG1 (#TA327295; Lot # 3515658101) and Rabbit polyclonal anti-SGK1 (#TA326894; Lot # 3561864002) were purchased from Origene. Rabbit polyclonal anti-Phospho-SGK1 (Thr256) (#44-1260G; Lot #2530313) was purchased from ThermoFisher Scientific. Secondary HRP Antibody Goat anti-Rabbit IgG (#074-1506; Lot # 10440068) was purchased from KPL, Secondary HRP Antibody Rabbit anti-Mouse (#61-6520; Lot # XC342755A) and Secondary HRP Antibody Goat anti-Rat (#31470; Lot # XG344754) were purchased from Invitrogen. All primary antibodies were used at a concentration of 1:1000, and secondary antibodies were used at a concentration of 1:5000. See Supplementary Table 6.

Validation

All primary and secondary antibodies were used following Manufacturer's instructions. Molecular size markers have been included on each of the western blots, and the molecular weights for each of the antibodies were validated as per manufacturer's datasheets. Specificity for RICTOR, RAPTOR and TSC1 antibodies was also validated using knock-out mouse models for each of the corresponding gene products. Specificity for MFF, DRPL, NDRG1, SGK1, AKT1/2, CDC42, OPA1 and MFNs antibodies was confirmed knock-down mouse models or knock-down cells for each of the corresponding gene products. Additionally, application validations can be obtained from the relevant vendor websites for the antibodies using the supplied catalog numbers.

Eukaryotic cell lines

Policy information about cell lines and Sex and Gender in Research

Cell line source(s)

NIH3T3 cells (CRL-1658), HepG2 cells (HB-8065) and AML12 cells (CRL-2254) were used in this study and purchased from ATCC. Isolation of embryonic fibroblasts from Rictor flox/flox mice was performed as described (Bio Protoc. 2013 Sep 20;3(18):e908).

Authentication

The cells used in this study were not independently authenticated by us.

Mycoplasma contamination

All experiments in this study were performed used mycoplasma-free cells. Cell culture medium was periodically subjected to PCR using REDTaq® ReadyMix™ PCR reaction mix (Sigma; R2523) for the detection of mycoplasma.

Commonly misidentified lines (See ICLAC register)	No misidentified cell lines were used in this study.

Animals and other research organisms

Policy information about studies involving animals; ARRIVE guidelines recommended for reporting animal research, and Sex and Gender in Research

Laboratory animals	C57BL/6 (#000664), Rictor flox/flox (#020649), Raptor flox/flox (#013188) and Tsc1 flox/flox (#005680) mice were purchased from the Jackson Laboratory. Studies were performed in 2-10-month-old male and female mice fed regular chow (5058; Lab Diet, St Louis, MO, USA) and maintained in barrier facility at 22-23 ºC under 40-60% humidity and a 12h:12h light/dark cycle.
Wild animals	No wild animals were use in this study.
Reporting on sex	Studies were performed in 2-8-month-old male and female mice.
Field-collected samples	No field-collected samples were used in this study.
Ethics oversight	This research complies with all relevant ethical regulations including animal protocol approval from the IACUC of Albert Einstein College of Medicine (Protocol Number: 00001051).

Note that full information on the approval of the study protocol must also be provided in the manuscript.
