# Supplementary material for: mTORC2–NDRG1–CDC42 axis couples fasting to mitochondrial fission
Source: Nat Cell Biol. 2023 Jun 29;25(7):989–1003. doi: 10.1038/s41556-023-01163-3 (PMC10344787; doi:10.1038/s41556-023-01163-3)

Uncropped full-length pictures of IB membranes

Fig 1h. P-P70^{Thr389}

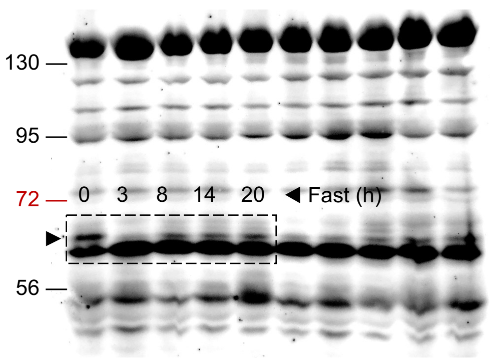

Fig 1h. P70

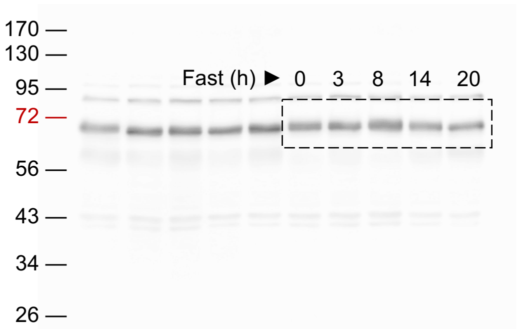

Fig 1h. P-S6$^{Ser235/236}$

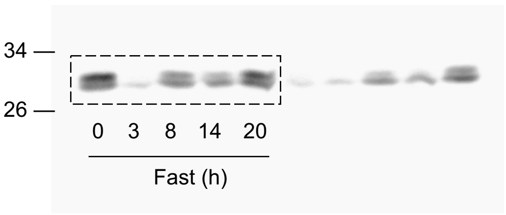

Fig 1h. S6

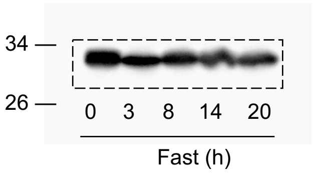

Fig 1h. P-AKT^{Ser473}

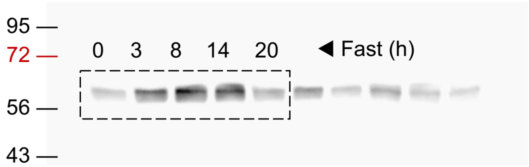

Fig 1h. P-AKT^{Thr308}

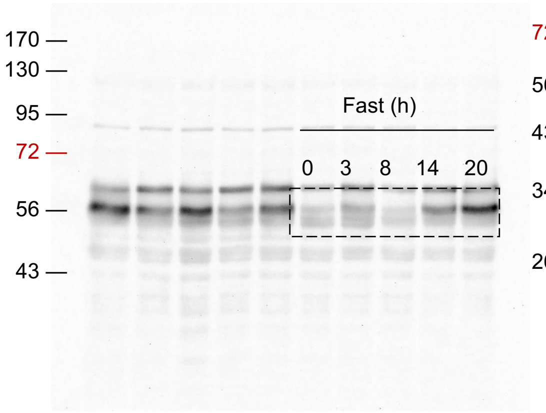

Fig 1h. AKT

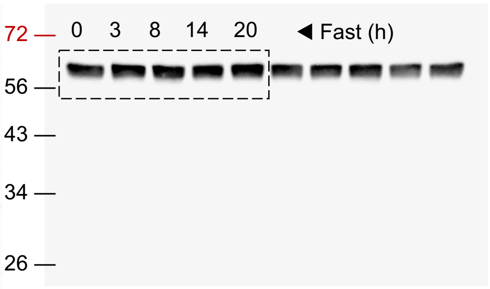

Fig 1h. P-SGK1^{Thr256}

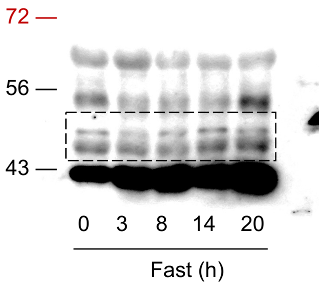

Fig 1h. SGK1

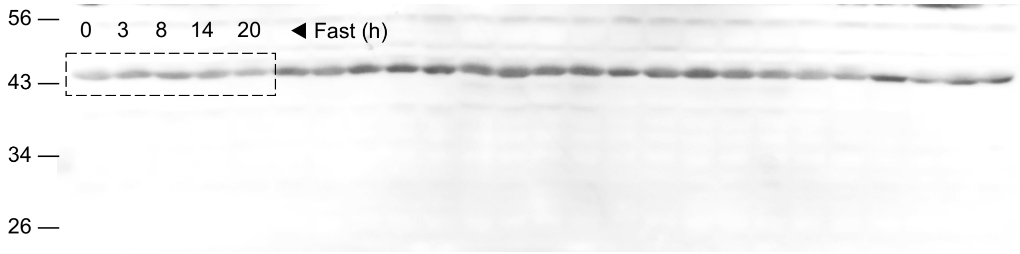

Fig 1h. P-NDRG1^{Thr346}

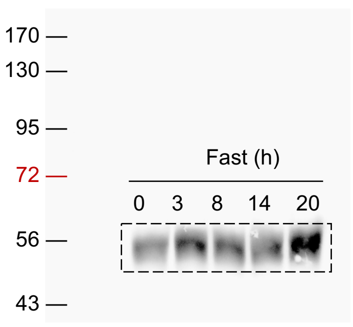

Fig 1h. NDRG1

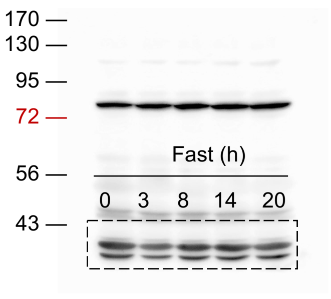

Fig 1h. Ponceau

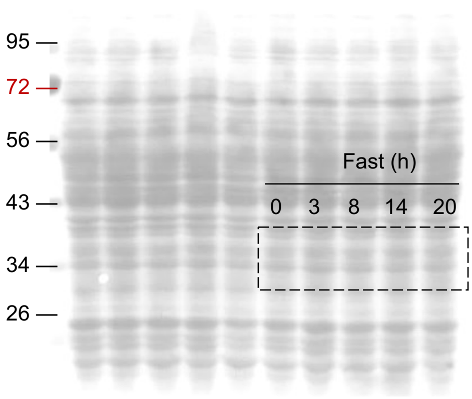

Supplement: Source Data Fig. 1 — Unprocessed western blots for Fig. 1. [file 41556_2023_1163_MOESM19_ESM.pdf]
