# Supplementary material for: mTORC2–NDRG1–CDC42 axis couples fasting to mitochondrial fission
Source: Nat Cell Biol. 2023 Jun 29;25(7):989–1003. doi: 10.1038/s41556-023-01163-3 (PMC10344787; doi:10.1038/s41556-023-01163-3)

Uncropped full-length pictures of IB membranes

Fig 3c. RICTOR

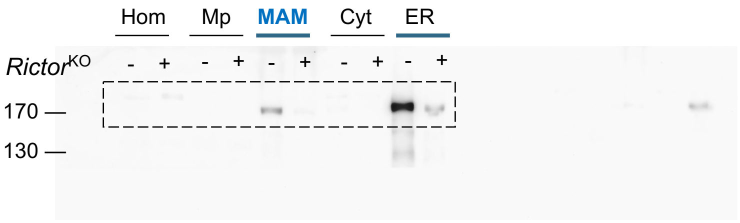

Fig 3c. MFN1

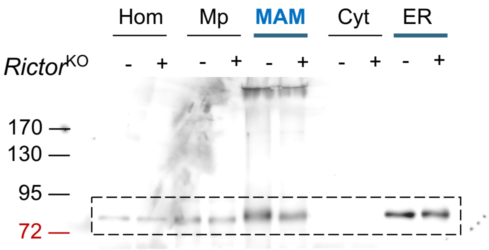

Fig 3c. MFN2

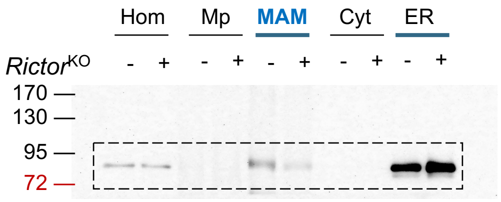

Fig 3c. OPA1

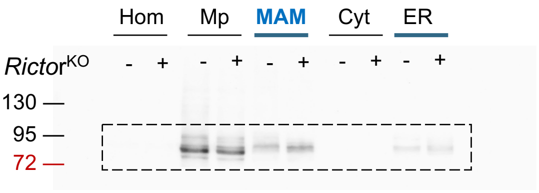

Fig 3c. P-DRP1^{Ser616}

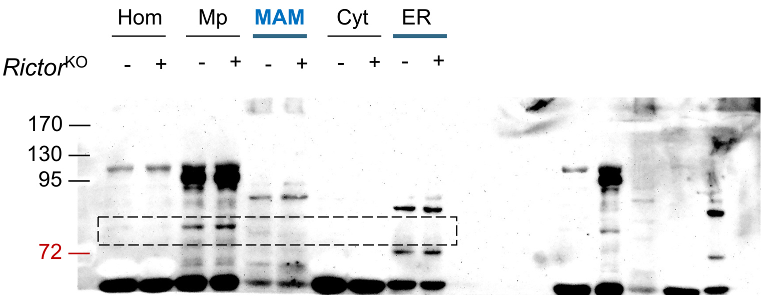

Fig 3c. P-DRP1^{Ser637}

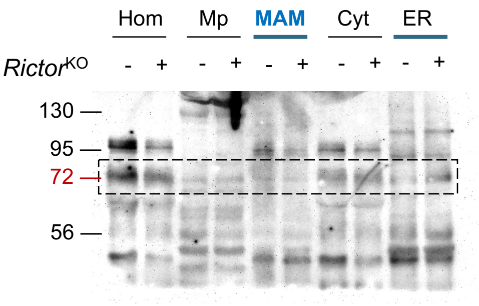

Fig 3c. DRP1

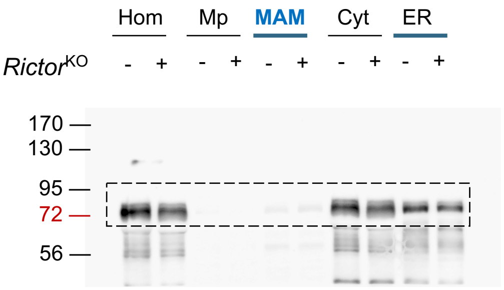

Fig 3c. MFF

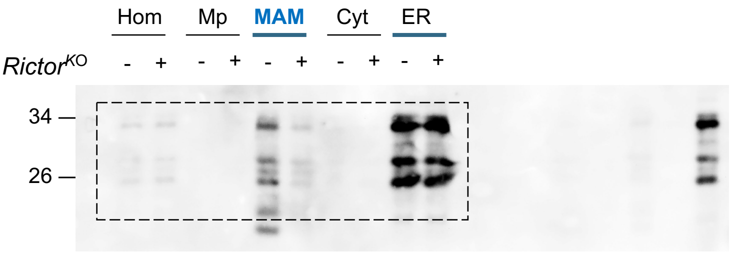

Fig 3c. CYT C

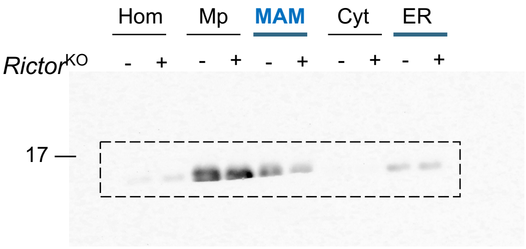

Fig 3c. PARKIN

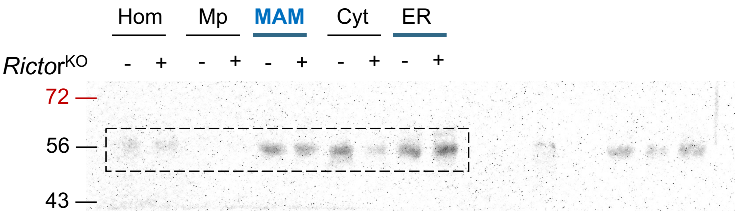

Fig 3c. CALRETICULIN

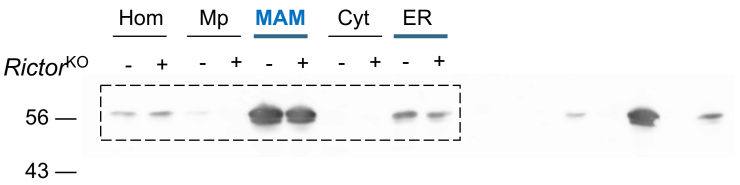

Fig 3c. Ponceau

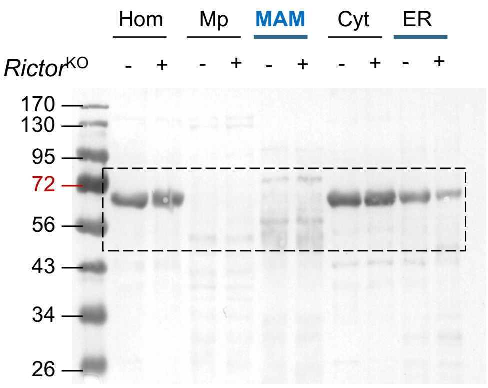

Fig 3c. FACL4

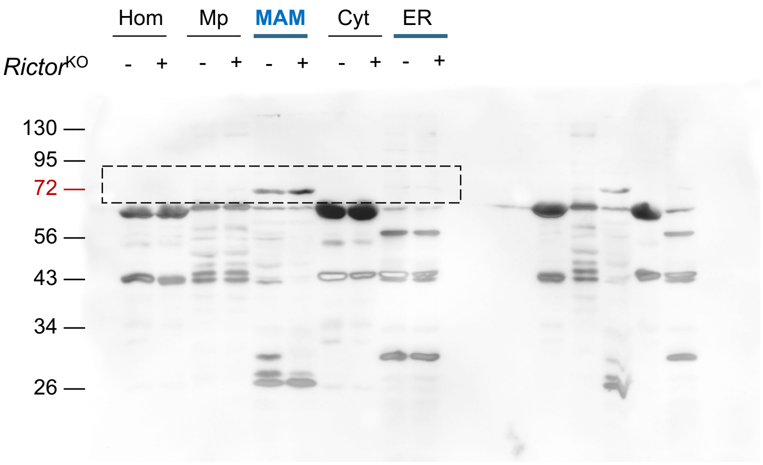

Fig 3c. TUBULIN

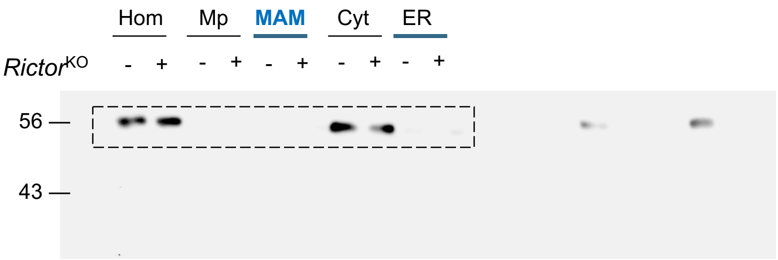

Fig 3e. DRP1

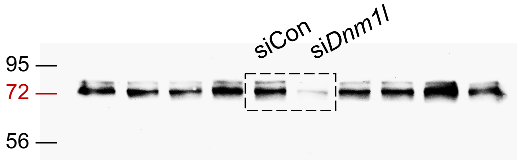

Fig 3e. RICTOR

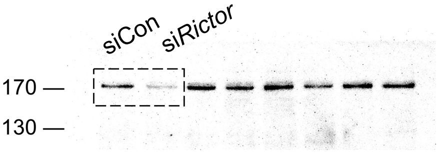

Fig 3e. Ponceau (si*Dnm1l*)

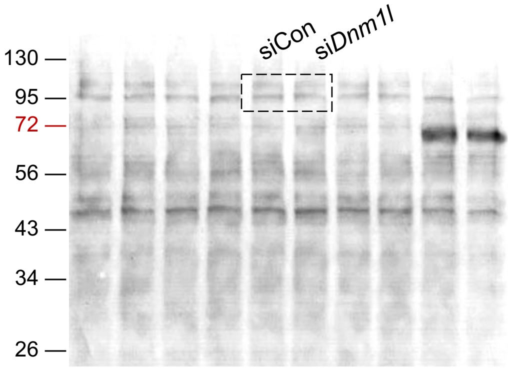

Fig 3e. Ponceau (si*Rictor*)

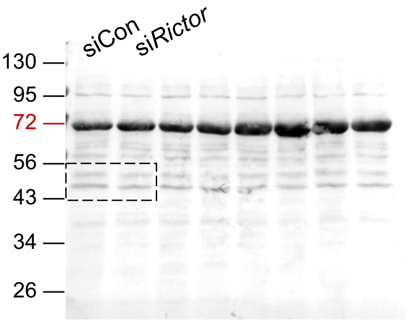

Supplement: Source Data Fig. 3 — Unprocessed western blots for Fig. 3. [file 41556_2023_1163_MOESM21_ESM.pdf]
