# Supplementary figures and images for: mTORC2–NDRG1–CDC42 axis couples fasting to mitochondrial fission
Source: Nat Cell Biol. 2023 Jun 29;25(7):989–1003. doi: 10.1038/s41556-023-01163-3 (PMC10344787; doi:10.1038/s41556-023-01163-3)

Uncropped full-length pictures of IB membranes

Fig 6b. FLAG

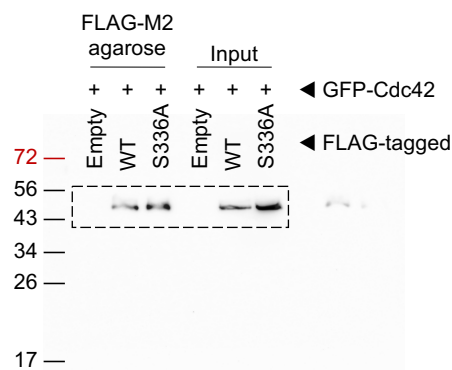

Fig 6b. GFP

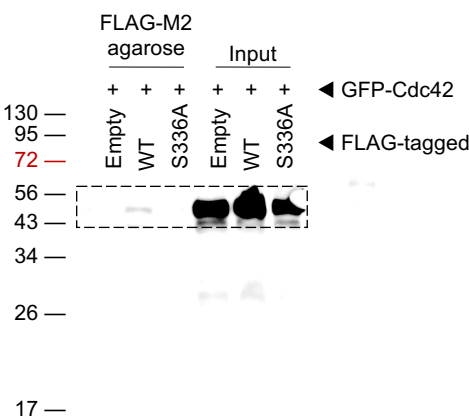

Fig 6b. Ponceau

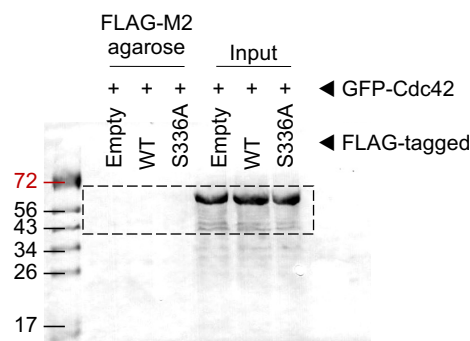

Supplement: Source Data Fig. 6 — Unprocessed western blots for Fig. 6. [file 41556_2023_1163_MOESM23_ESM.pdf]
