# Supplementary material for: mTORC2–NDRG1–CDC42 axis couples fasting to mitochondrial fission
Source: Nat Cell Biol. 2023 Jun 29;25(7):989–1003. doi: 10.1038/s41556-023-01163-3 (PMC10344787; doi:10.1038/s41556-023-01163-3)

Uncropped full-length pictures of IB membranes

Fig 7d. CDC42

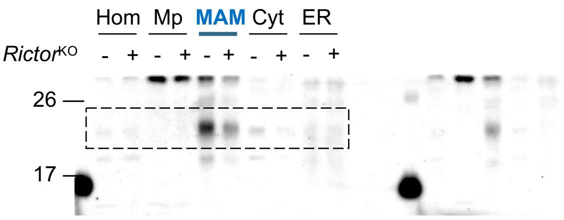

Fig 7d. RHOA

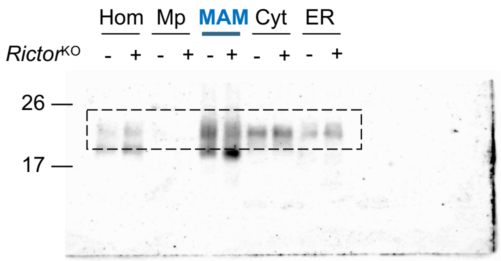

Fig 7d. DYNAMIN I/II

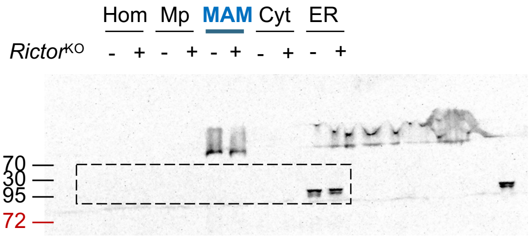

Fig 7d. CYT C

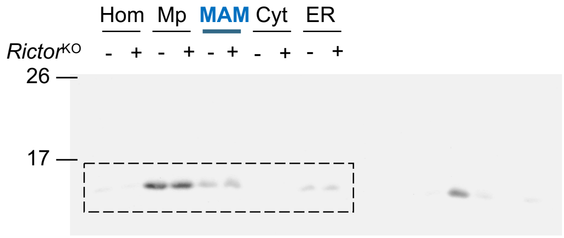

Fig 7d. FACL4

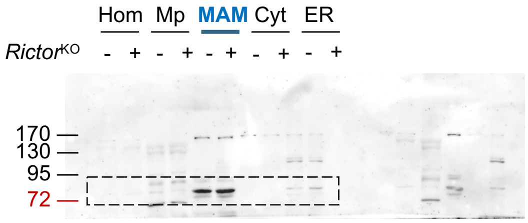

Fig 7d. CALRETICULIN

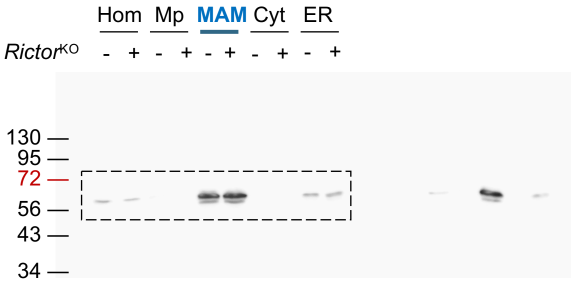

Fig 7d. TUBULIN

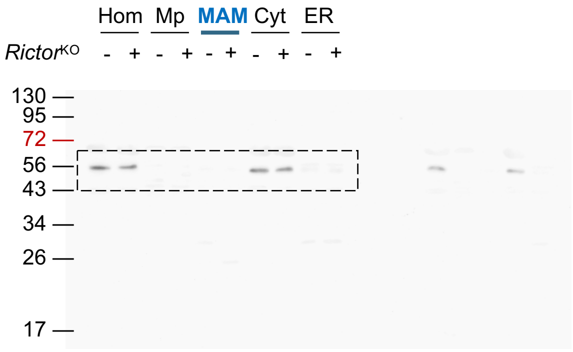

Fig 7d. Ponceau

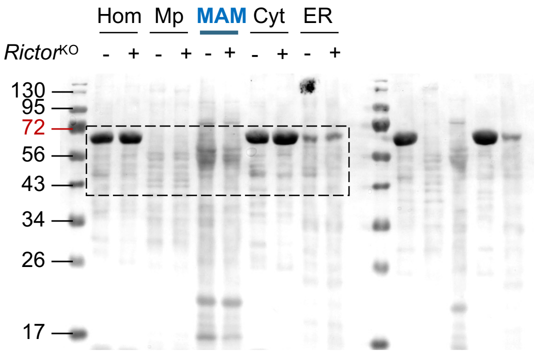

Fig 7e. CDC42

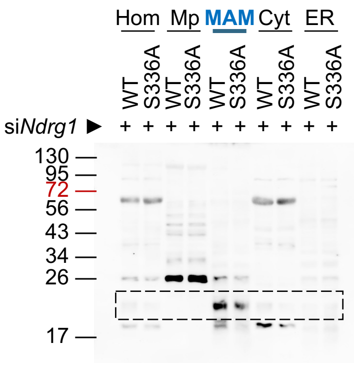

Fig 7e. RHOA

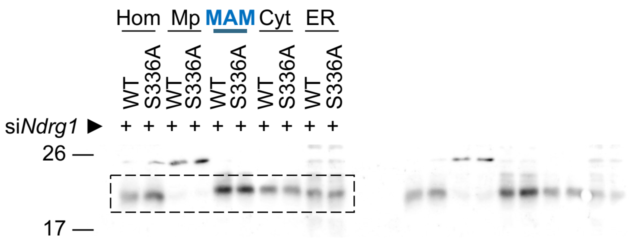

Fig 7e. VDAC1

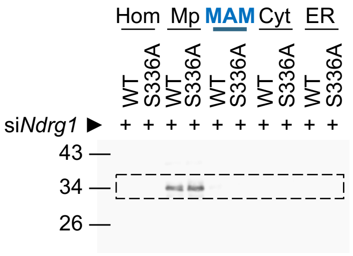

Fig 7e. FACL4

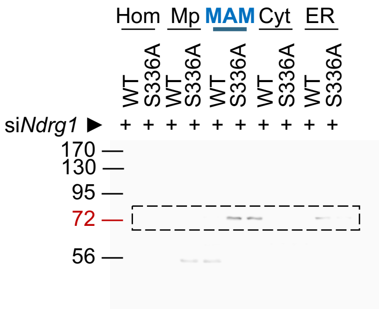

Fig 7e. CALRETICULIN

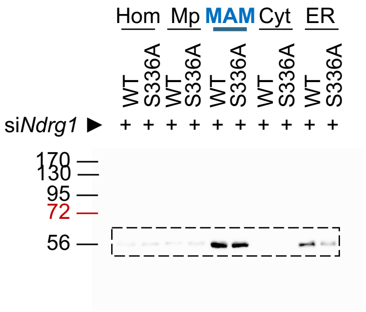

Fig 7e. TUBULIN

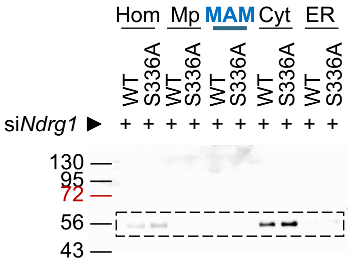

Fig 7e. Ponceau

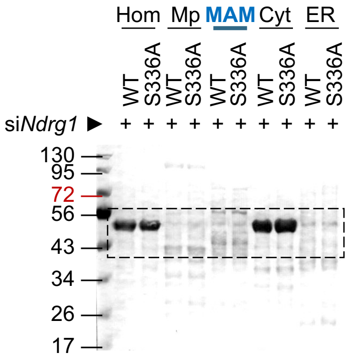

Supplement: Source Data Fig. 7 — Unprocessed western blots for Fig. 7. [file 41556_2023_1163_MOESM24_ESM.pdf]
