# Supplementary material for: mTORC2–NDRG1–CDC42 axis couples fasting to mitochondrial fission
Source: Nat Cell Biol. 2023 Jun 29;25(7):989–1003. doi: 10.1038/s41556-023-01163-3 (PMC10344787; doi:10.1038/s41556-023-01163-3)

Uncropped full-length pictures of IB membranes

Extended Data 1e. RAPTOR

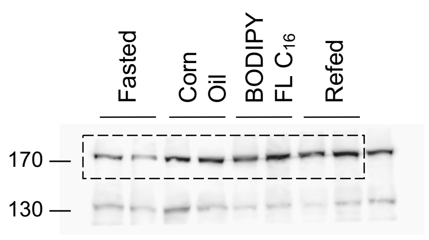

Extended Data 1e. RICTOR

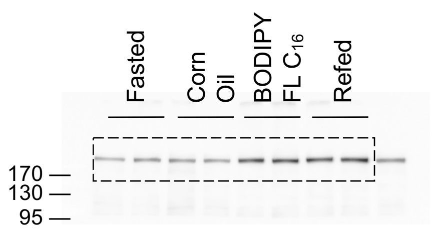

Extended Data 1e. P-P70^{Thr389}

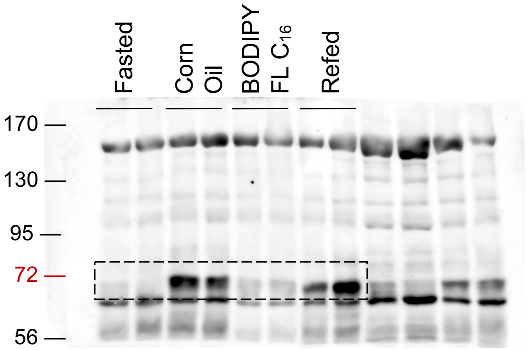

Extended Data 1e. P70

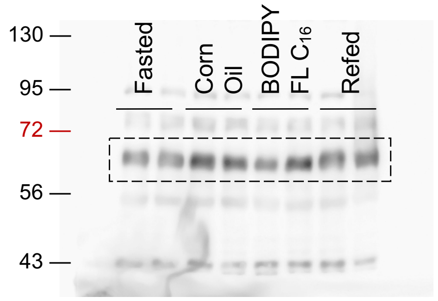

Extended Data 1e. P-S6$^{Ser235/236}$

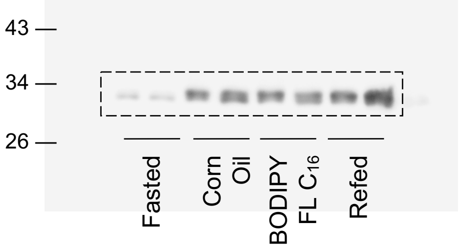

Extended Data 1e. S6

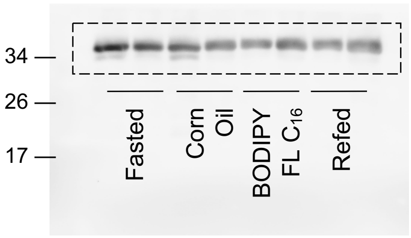

Extended Data 1e. P-AKT^{Thr308}

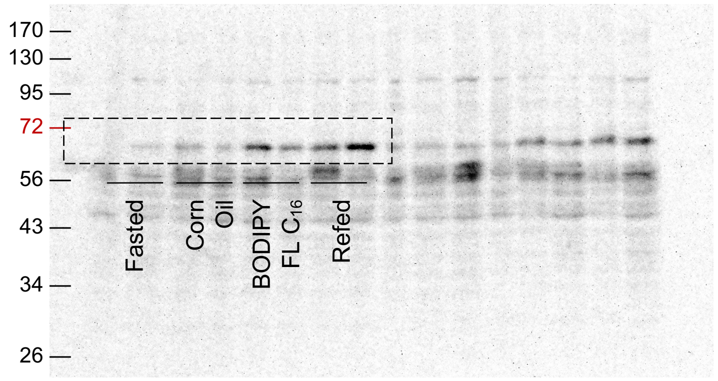

Extended Data 1e. P-AKT^{Ser473}

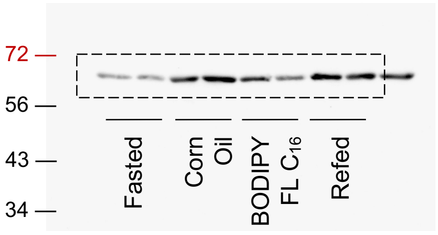

Extended Data 1e. AKT

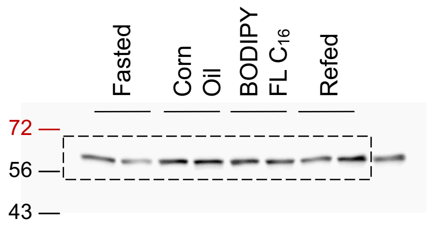

Extended Data 1e. P-AMPKα^{Thr172}

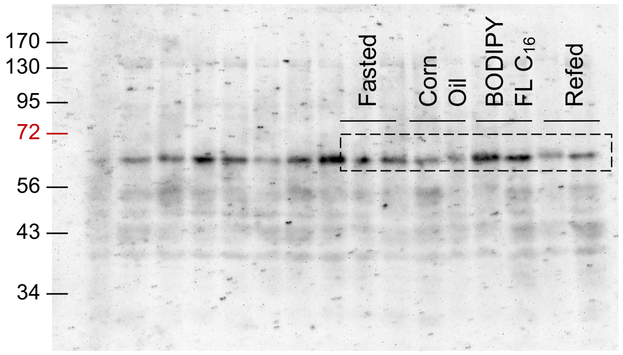

Extended Data 1e. Ponceau

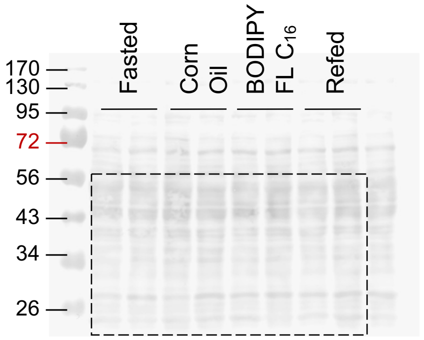

Extended Data 1e. AMPKα

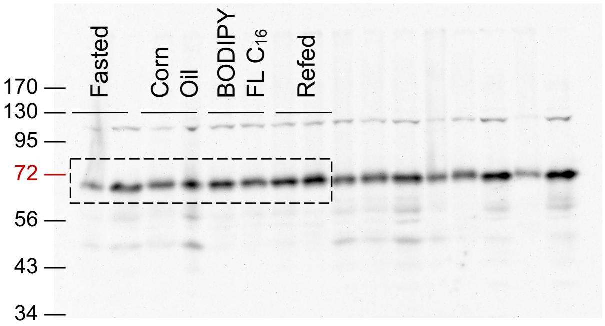

Supplement: Source Data Extended Data Fig. 1 — Unprocessed western blots for Extended Data Fig. 1. [file 41556_2023_1163_MOESM25_ESM.pdf]
