# Supplementary material for: mTORC2–NDRG1–CDC42 axis couples fasting to mitochondrial fission
Source: Nat Cell Biol. 2023 Jun 29;25(7):989–1003. doi: 10.1038/s41556-023-01163-3 (PMC10344787; doi:10.1038/s41556-023-01163-3)

Uncropped full-length pictures of IB membranes

Extended Data Fig 2d. P-S6$^{Ser235/236}$

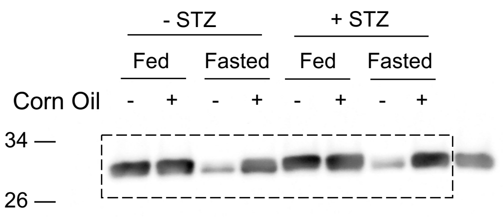

Extended Data Fig 2d. S6

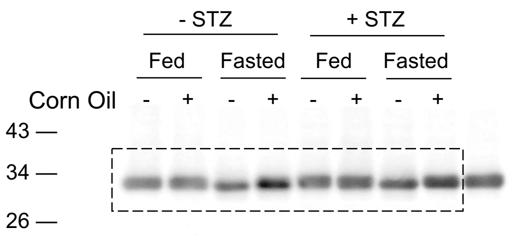

Extended Data Fig 2d. P-AKT^{Ser473}

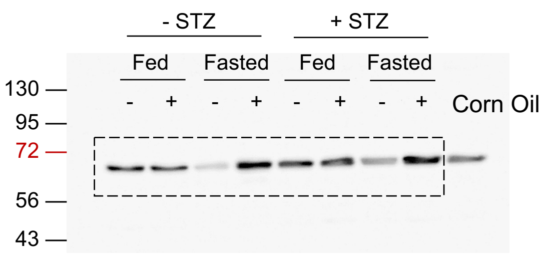

Extended Data Fig 2d. P-AKT^{Thr308}

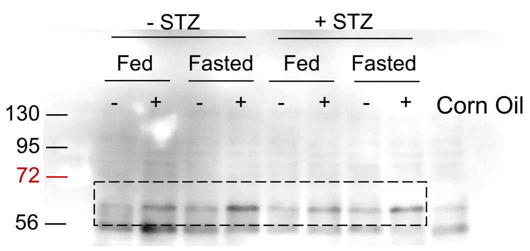

Extended Data Fig 2d. AKT

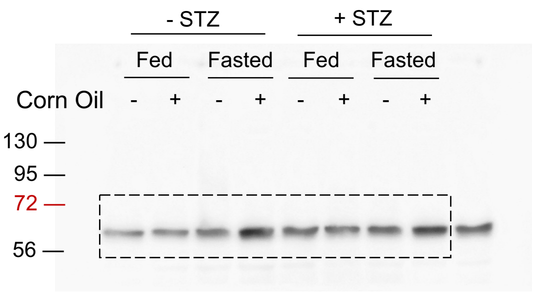

Extended Data Fig 2d. Ponceau

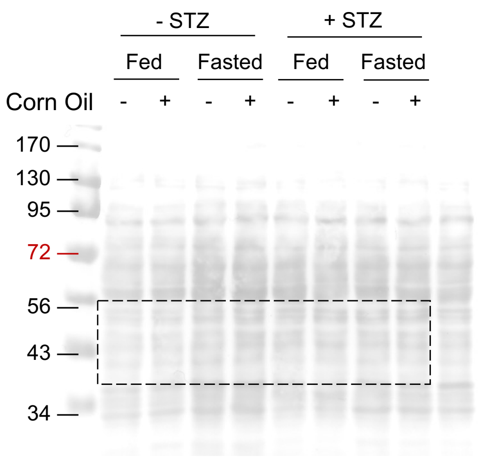

Extended Data Fig 2j. P-PKA^{Thr197}

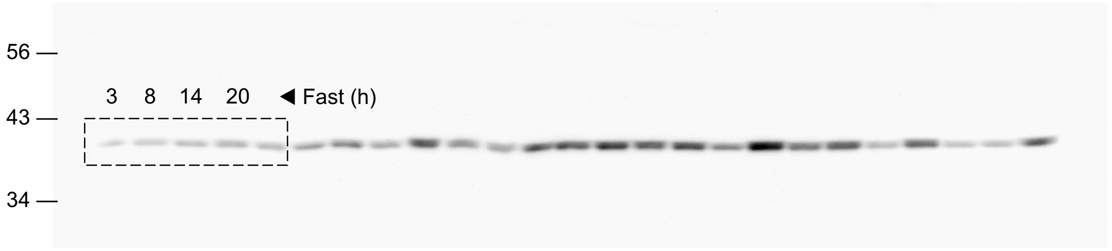

Extended Data Fig 2j. PKA

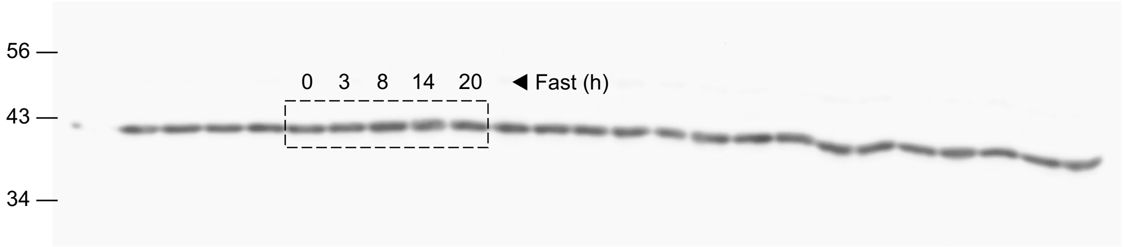

Extended Data Fig 2j. P-PKCα/βII$^{Thr638/641}$

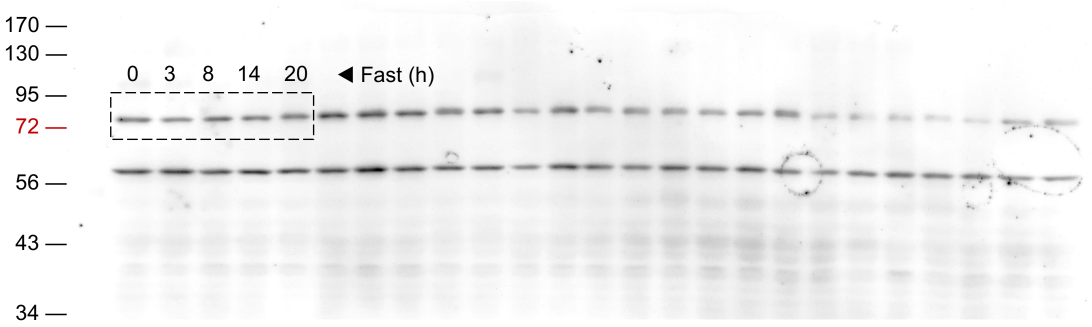

Extended Data Fig 2j. PKCα

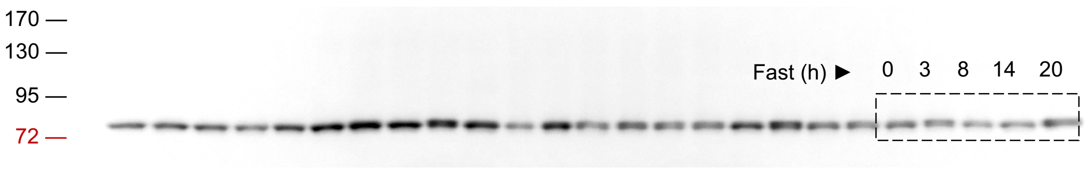

Extended Data Fig 2j. P-PKCδ^{Thr505}

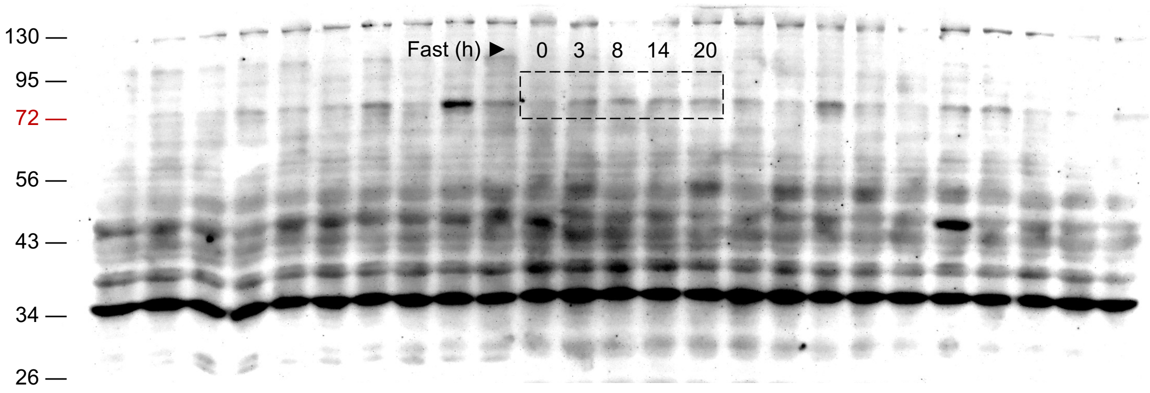

Extended Data Fig 2j. P-PKCδ/θ$^{S643/676}$

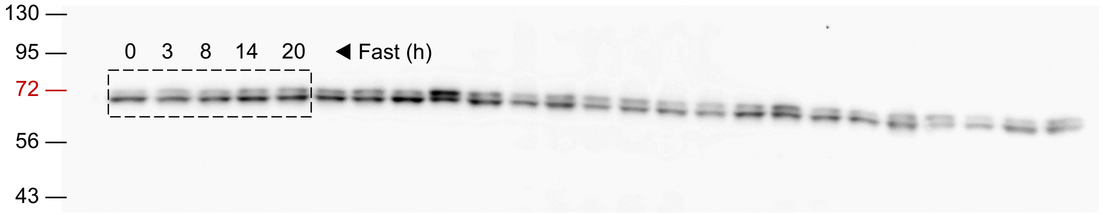

Extended Data Fig 2j. PKCδ

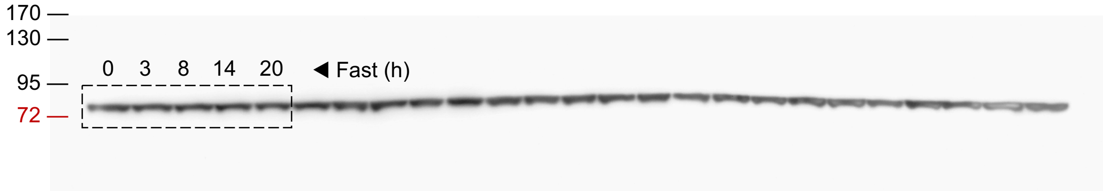

Extended Data Fig 2j. P-PKCζ/λ$^{Thr410/403}$

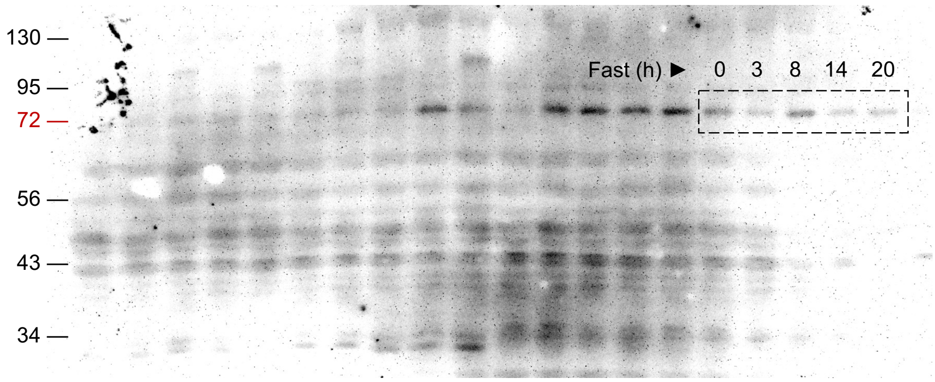

Extended Data Fig 2j. PKCζ

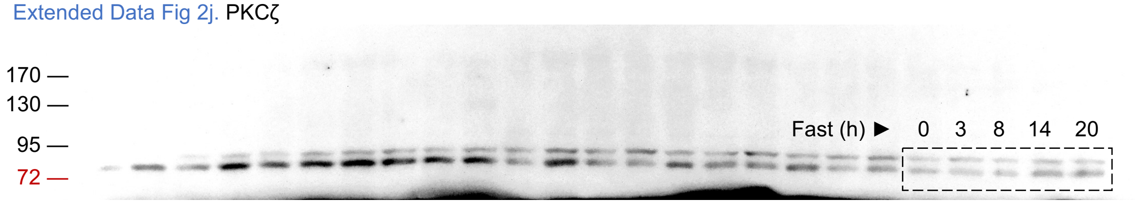

Extended Data Fig 2j. Ponceau

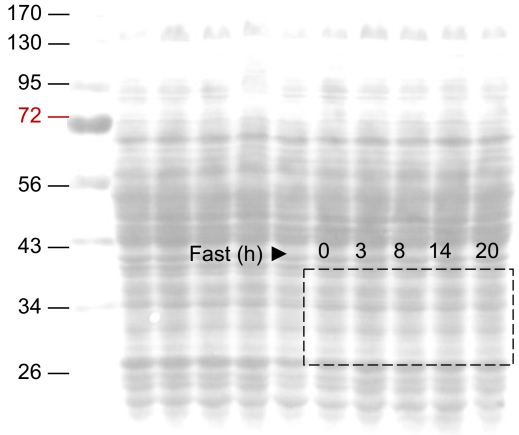

Supplement: Source Data Extended Data Fig. 2 — Unprocessed western blots for Extended Data Fig. 2. [file 41556_2023_1163_MOESM26_ESM.pdf]
