# Supplementary material for: mTORC2–NDRG1–CDC42 axis couples fasting to mitochondrial fission
Source: Nat Cell Biol. 2023 Jun 29;25(7):989–1003. doi: 10.1038/s41556-023-01163-3 (PMC10344787; doi:10.1038/s41556-023-01163-3)

Uncropped full-length pictures of IB membranes

Extended Data Fig 3a. TSC1

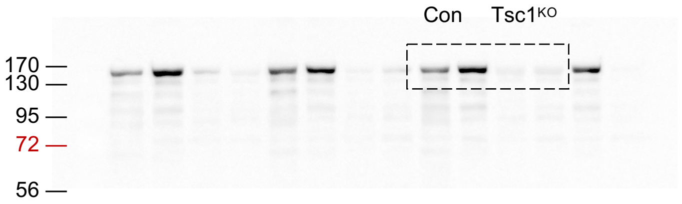

Extended Data Fig 3b. RAPTOR

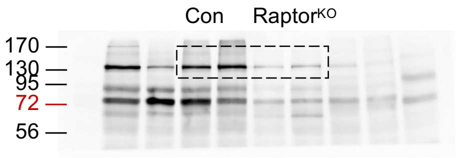

Extended Data Fig 3a. Ponceau (TSC1)

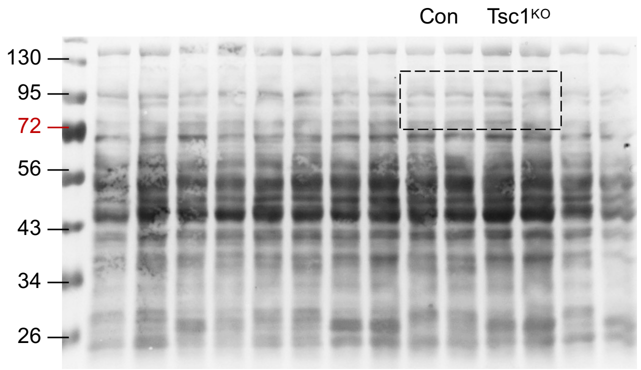

Extended Data Fig 3b. Ponceau (RAPTOR)

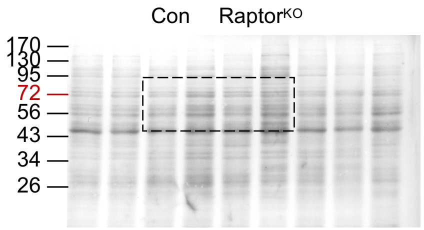

Extended Data Fig 3c. RICTOR (Liver)

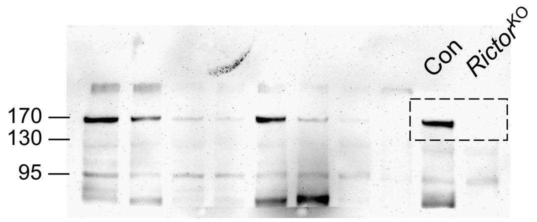

Extended Data Fig 3c. P-AKTSer473 (Liver)

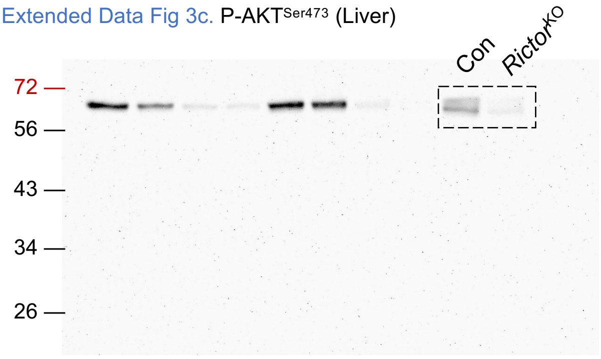

Extended Data Fig 3c. AKT (Liver)

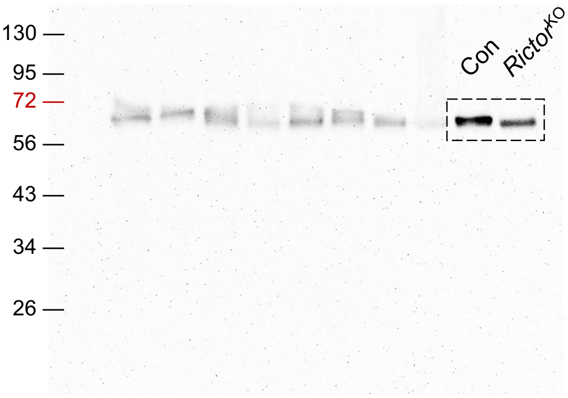

Extended Data Fig 3c. Ponceau (Liver)

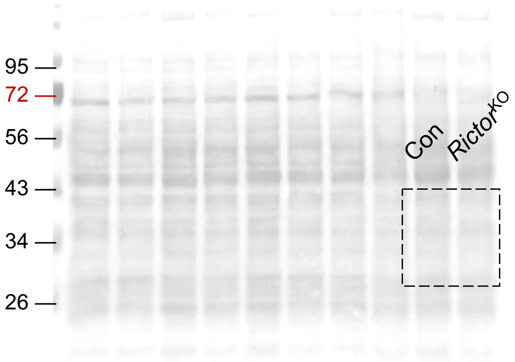

Extended Data Fig 3c. RICTOR (eWAT)

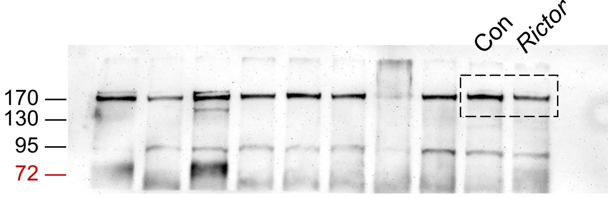

Extended Data Fig 3c. AKT (eWAT)

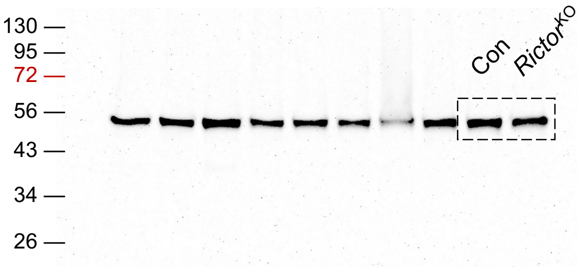

Extended Data Fig 3c. P-AKT^{Ser473} (eWAT)

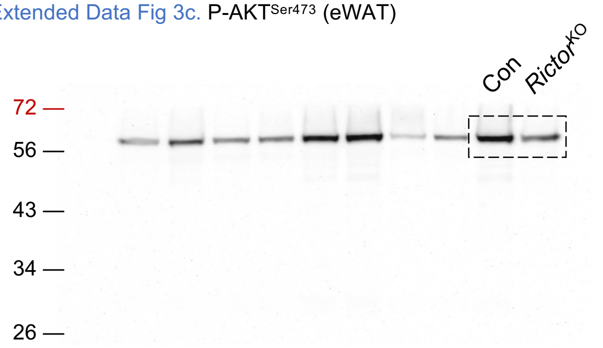

Extended Data Fig 3c. Ponceau (eWAT)

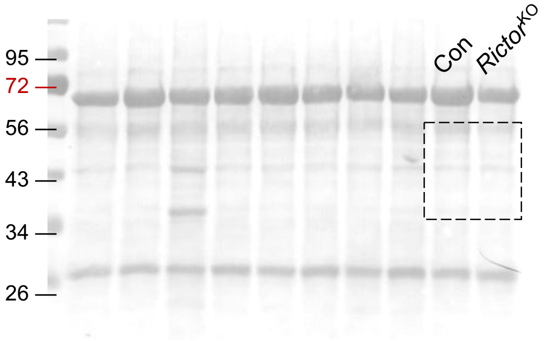

Extended Data Fig 3c. RICTOR (Soleus)

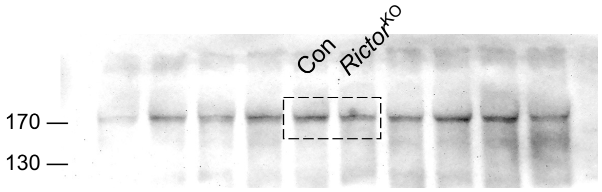

Extended Data Fig 3c. P-AKT^{Ser473} (Soleus)

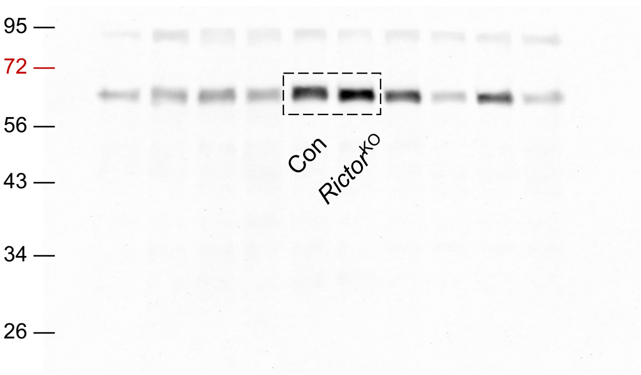

Extended Data Fig 3c. AKT (Soleus)

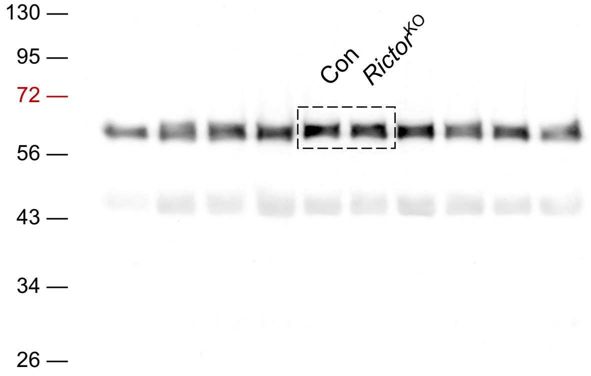

Extended Data Fig 3c. Ponceau (Soleus)

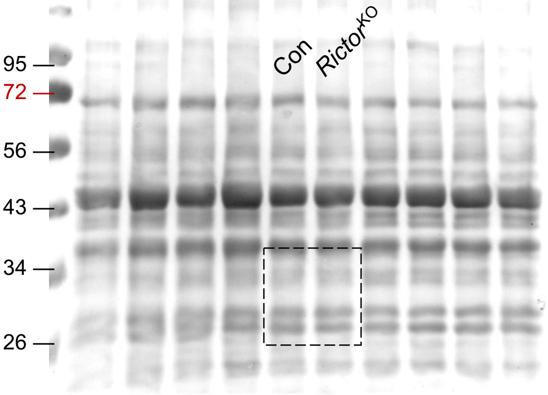

Extended Data Fig 3t. CPT1A

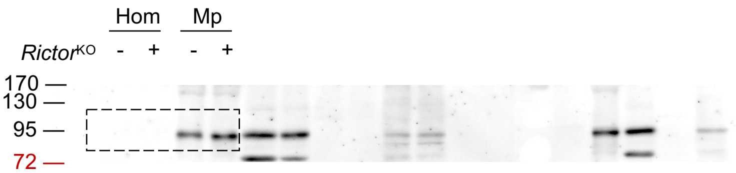

Extended Data Fig 3t. CACT

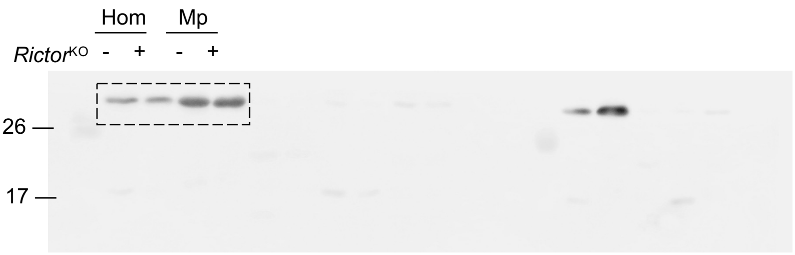

Extended Data Fig 3t. CPT2

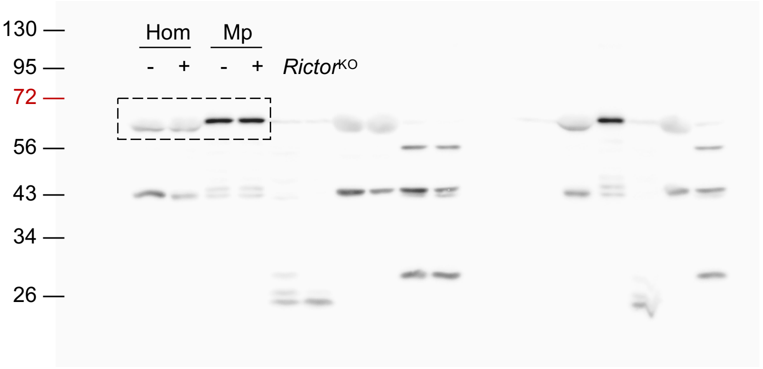

Extended Data Fig Fig 3t. Ponceau

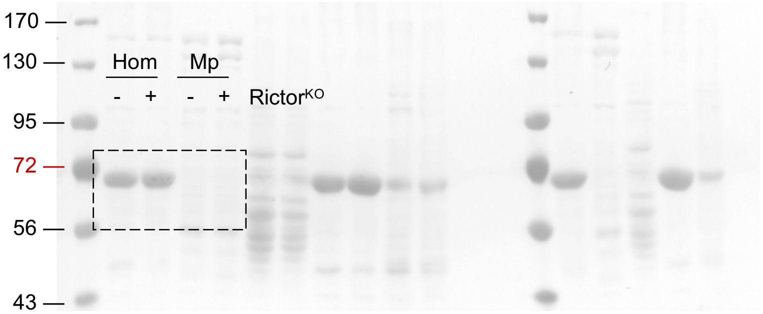

Extended Data Fig 3u. OXPHOS

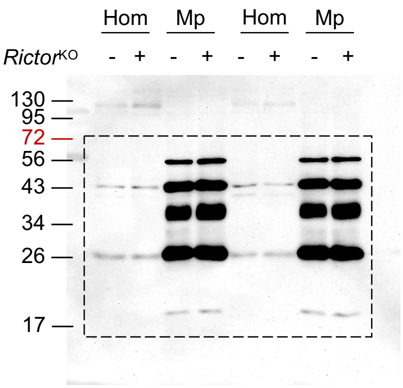

Extended Data Fig 3u. Ponceau

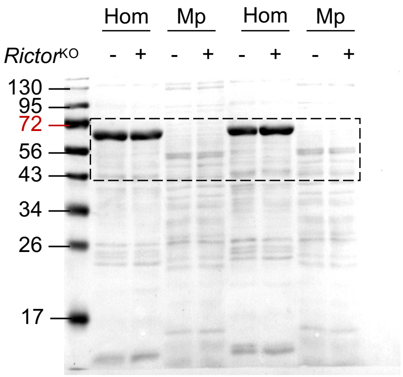

Supplement: Source Data Extended Data Fig. 3 — Unprocessed western blots for Extended Data Fig. 3. [file 41556_2023_1163_MOESM27_ESM.pdf]
