# Supplementary material for: mTORC2–NDRG1–CDC42 axis couples fasting to mitochondrial fission
Source: Nat Cell Biol. 2023 Jun 29;25(7):989–1003. doi: 10.1038/s41556-023-01163-3 (PMC10344787; doi:10.1038/s41556-023-01163-3)

Uncropped full-length pictures of IB membranes

Extended Data Fig 4d. VDAC1

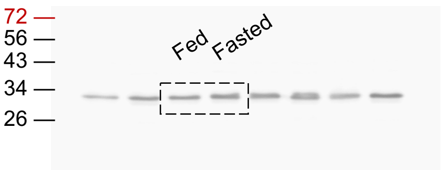

Extended Data Fig 4d. CYT C

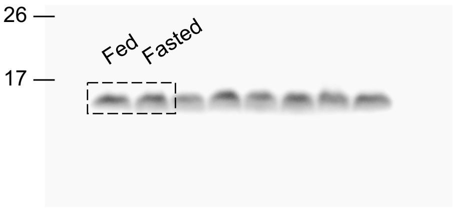

Extended Data Fig 4d. Ponceau

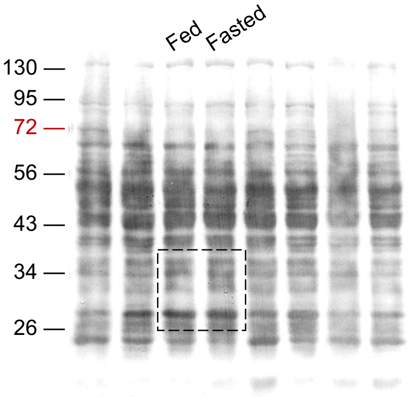

Supplement: Source Data Extended Data Fig. 4 — Unprocessed western blots for Extended Data Fig. 4. [file 41556_2023_1163_MOESM28_ESM.pdf]
