# Supplementary material for: mTORC2–NDRG1–CDC42 axis couples fasting to mitochondrial fission
Source: Nat Cell Biol. 2023 Jun 29;25(7):989–1003. doi: 10.1038/s41556-023-01163-3 (PMC10344787; doi:10.1038/s41556-023-01163-3)

Uncropped full-length pictures of IB membranes

Extended Data Fig 5b. MFF

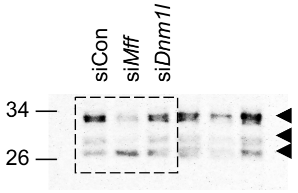

Extended Data Fig 5c. MFF

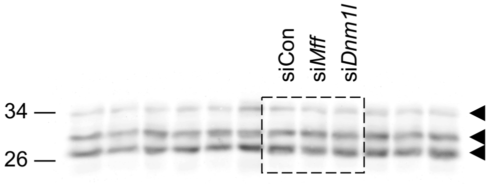

Extended Data Fig 5b. DRP1

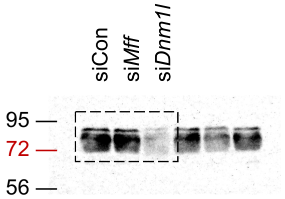

Extended Data Fig 5c. DRP1

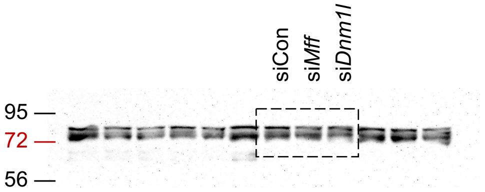

Extended Data Fig 5b. Ponceau

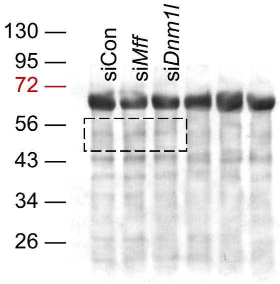

Extended Data Fig 5c. Ponceau

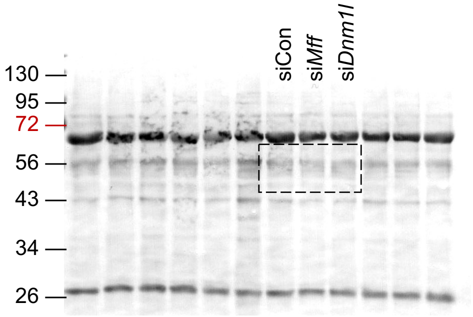

Extended Data Fig 5e. BIP

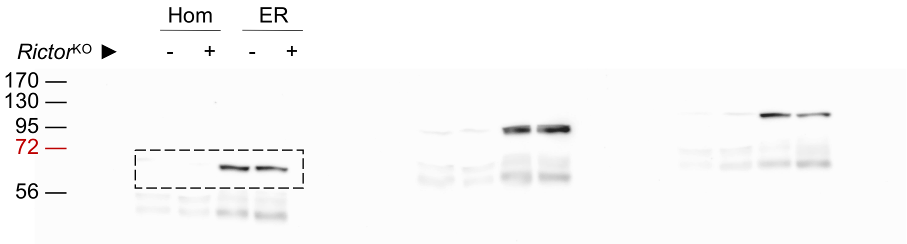

Extended Data Fig 5e. P-eIF2α^{Ser51}

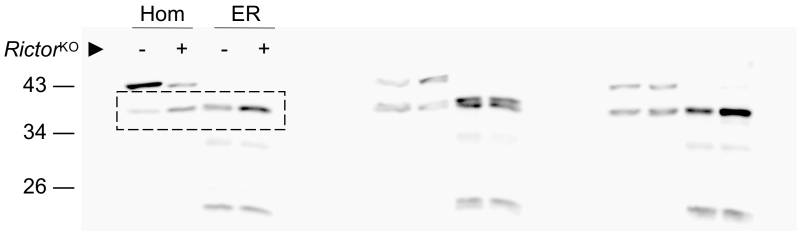

Extended Data Fig 5e. eIF2α

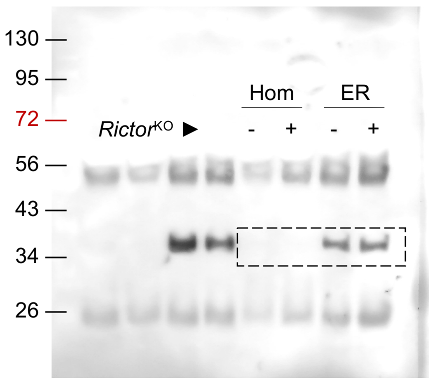

Extended Data Fig 5e. CHOP

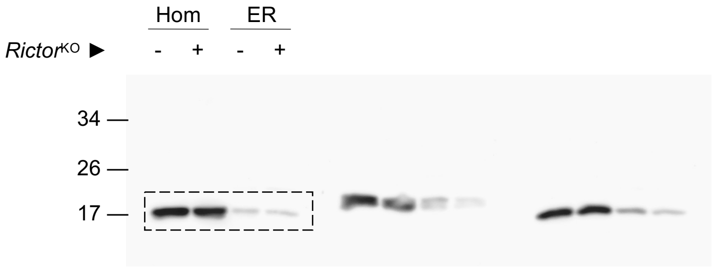

Extended Data Fig 5e. XBP-1s

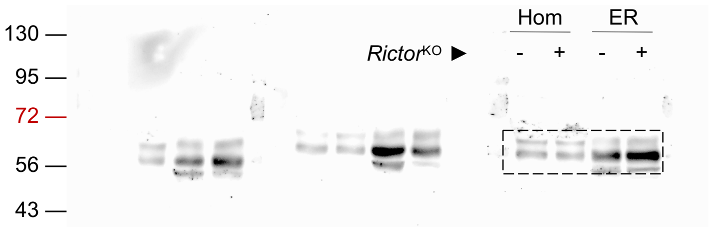

Extended Data Fig 5e. K48-linkage Specific Polyubiquitin

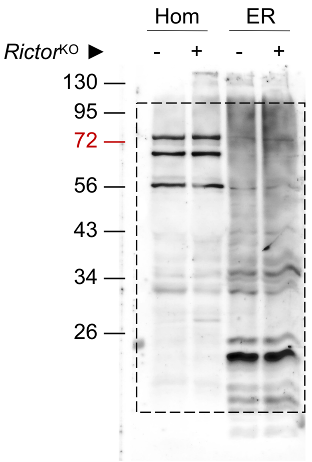

Extended Data Fig 5e. Ponceau

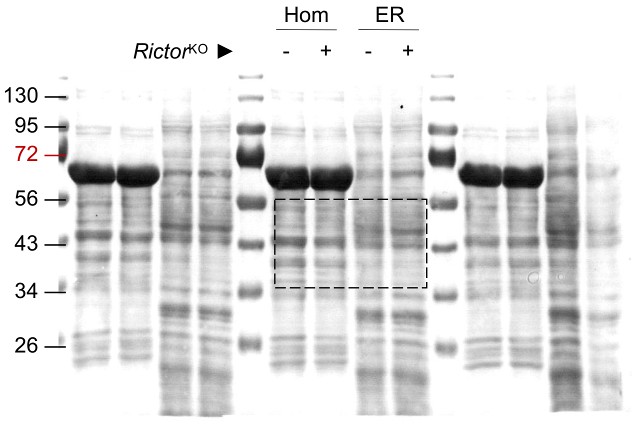

Extended Data Fig 5f. RICTOR

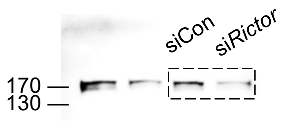

Extended Data Fig 5g. RICTOR

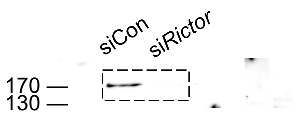

Extended Data Fig 5h. RICTOR

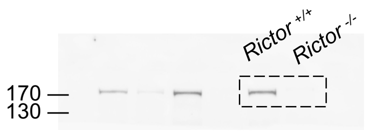

Extended Data Fig 5f. Ponceau

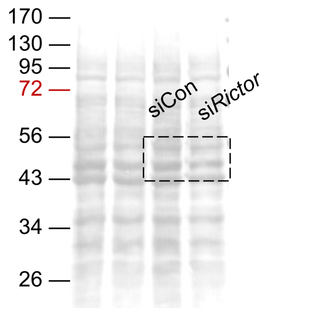

Extended Data Fig 5g. Ponceau

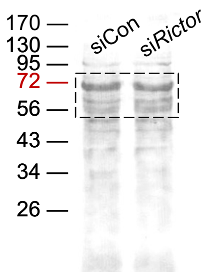

Extended Data Fig 5h. Ponceau

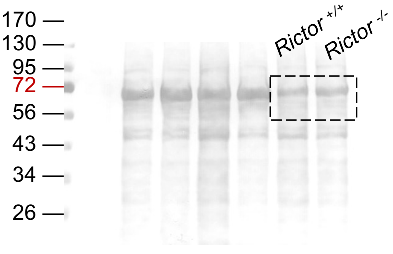

Extended Data 5j. P-AKT^{Ser473}

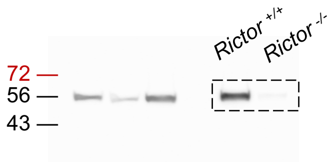

Extended Data 5j. AKT

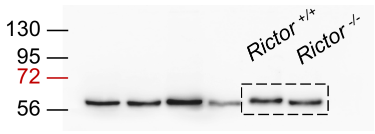

Extended Data 5j. P-NDRG1^{Thr346}

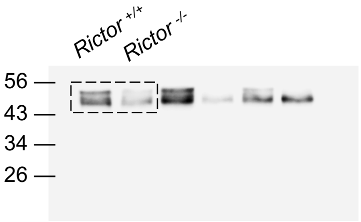

Extended Data 5j. NDRG1

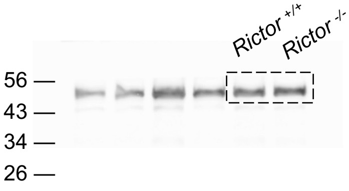

Extended Data 5j. P-DRP1^{Ser616}

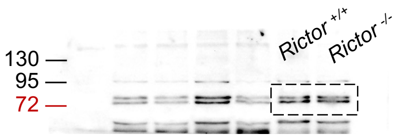

Extended Data 5j. P-DRP1^{Ser637}

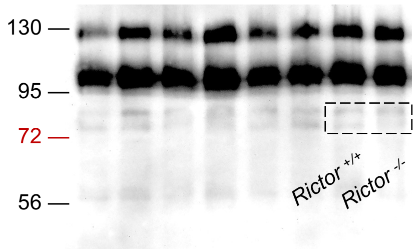

Extended Data 5j. DRP1

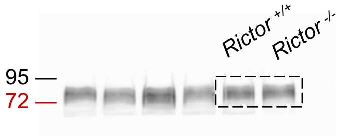

Supplement: Source Data Extended Data Fig. 5 — Unprocessed western blots for Extended Data Fig. 5. [file 41556_2023_1163_MOESM29_ESM.pdf]
