# Supplementary material for: mTORC2–NDRG1–CDC42 axis couples fasting to mitochondrial fission
Source: Nat Cell Biol. 2023 Jun 29;25(7):989–1003. doi: 10.1038/s41556-023-01163-3 (PMC10344787; doi:10.1038/s41556-023-01163-3)

Uncropped full-length pictures of IB membranes

Extended Data Fig 6g. NDRG1

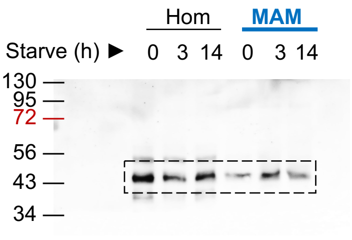

Extended Data Fig 6g. CALRETICULIN

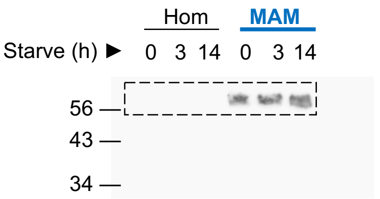

Extended Data Fig 6g. FACL4

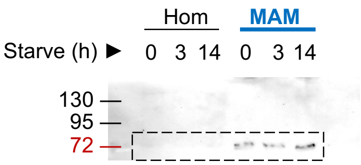

Extended Data Fig 6g. TUBULIN

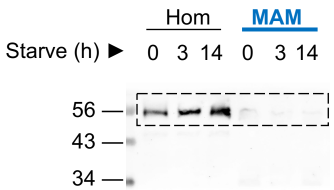

Extended Data Fig 6g. Ponceau

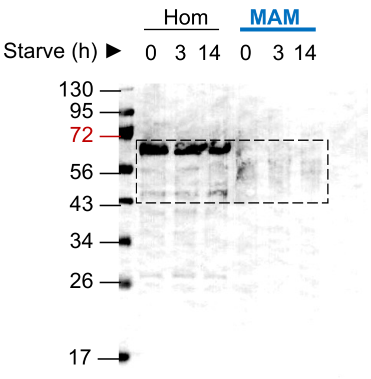

Supplement: Source Data Extended Data Fig. 6 — Unprocessed western blots for Extended Data Fig. 6. [file 41556_2023_1163_MOESM30_ESM.pdf]
