# Supplementary material for: mTORC2–NDRG1–CDC42 axis couples fasting to mitochondrial fission
Source: Nat Cell Biol. 2023 Jun 29;25(7):989–1003. doi: 10.1038/s41556-023-01163-3 (PMC10344787; doi:10.1038/s41556-023-01163-3)

Uncropped full-length pictures of IB membranes

Extended Data Fig 8b. SGK1

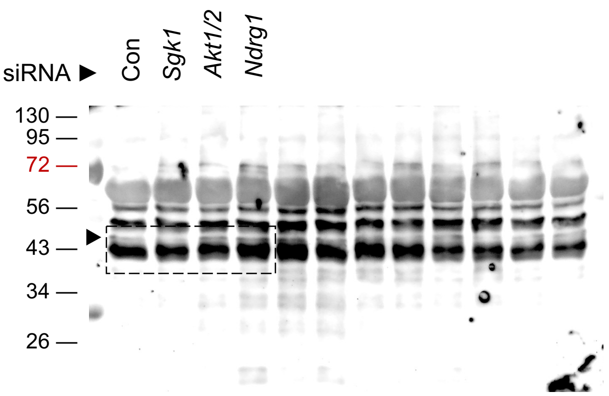

Extended Data Fig 8b. AKT1

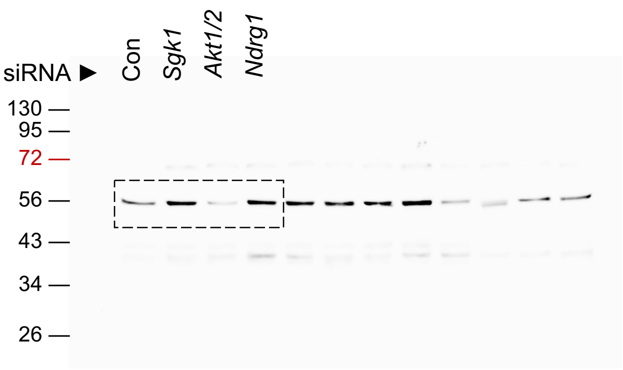

Extended Data Fig 8b.. AKT2

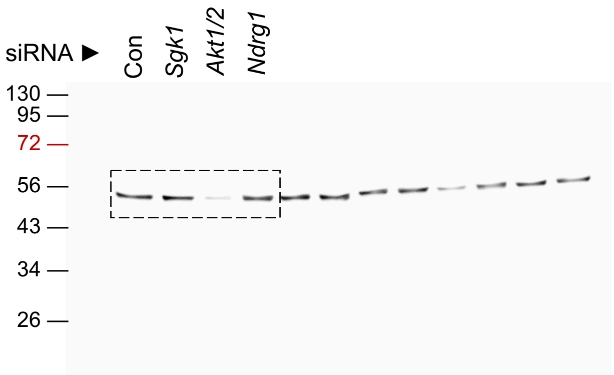

Extended Data Fig 8b. NDRG1 (rotated horizontally for the figure)

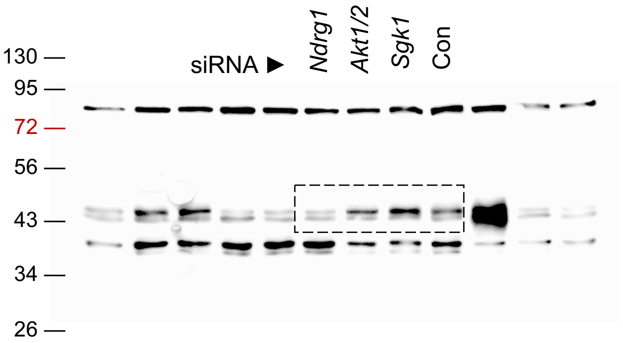

Extended Data Fig 8b. P-NDRG1Thr346

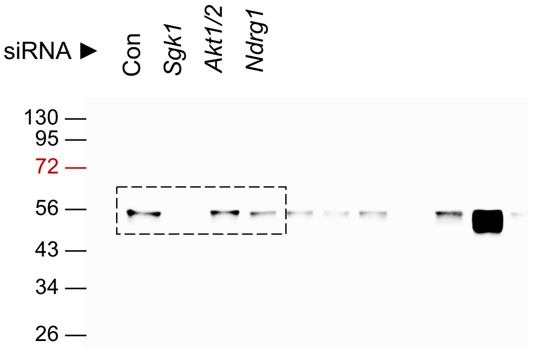

Extended Data Fig 8b. Ponceau

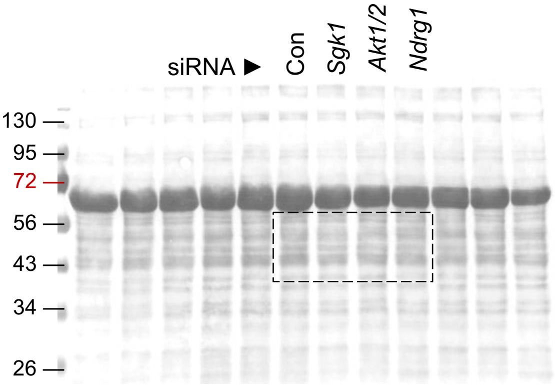

Supplement: Source Data Extended Data Fig. 8 — Unprocessed western blots for Extended Data Fig. 8. [file 41556_2023_1163_MOESM32_ESM.pdf]
