# Supplementary material for: mTORC2–NDRG1–CDC42 axis couples fasting to mitochondrial fission
Source: Nat Cell Biol. 2023 Jun 29;25(7):989–1003. doi: 10.1038/s41556-023-01163-3 (PMC10344787; doi:10.1038/s41556-023-01163-3)

Uncropped full-length pictures of IB membranes

Extended Data Fig 9a. DRP1

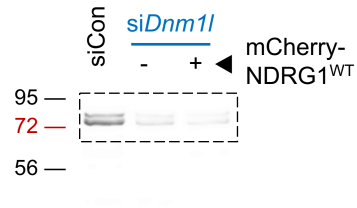

Extended Data Fig 9a. mCherry-NDRG1

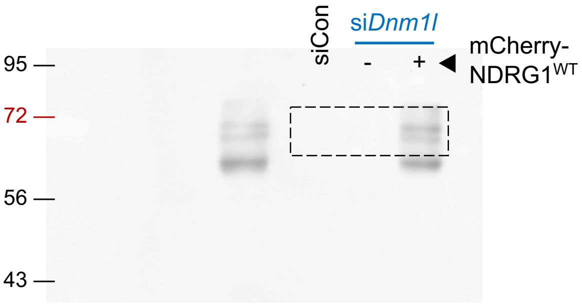

Extended Data Fig 9a. Ponceau

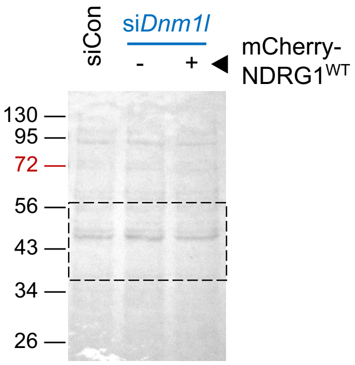

Extended Data Fig 9b. MFF

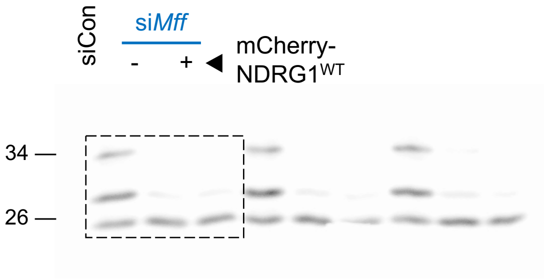

Extended Data Fig 9b. mCherry-NDRG1

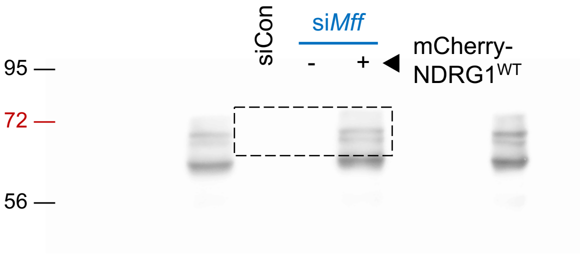

Extended Data Fig 9b. Ponceau

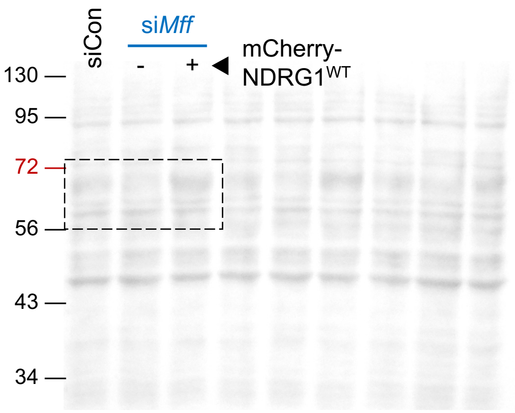

Extended Data Fig 9c. FLAG

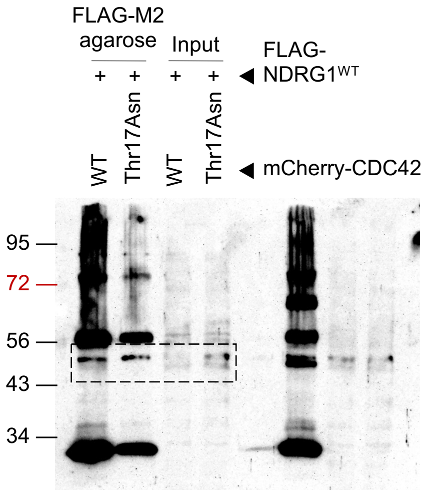

Extended Data Fig 9c. mCherry-Cdc42

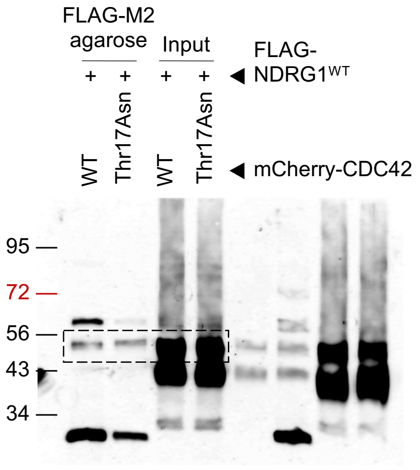

Extended Data Fig 9c. Ponceau

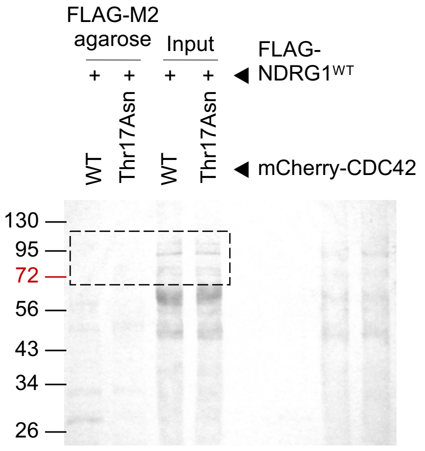

Extended Data Fig 9d. CDC42

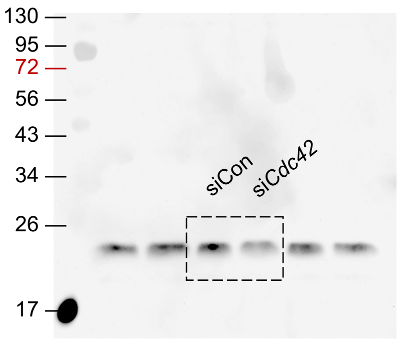

Extended Data Fig 9d. Ponceau

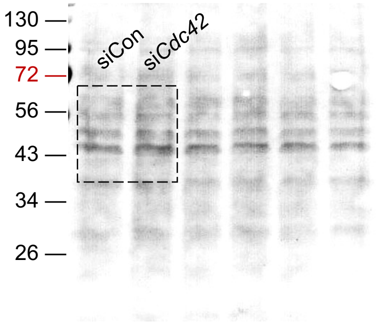

Supplement: Source Data Extended Data Fig. 9 — Unprocessed western blots for Extended Data Fig. 9. [file 41556_2023_1163_MOESM33_ESM.pdf]
