# Supplementary material for: mTORC2–NDRG1–CDC42 axis couples fasting to mitochondrial fission
Source: Nat Cell Biol. 2023 Jun 29;25(7):989–1003. doi: 10.1038/s41556-023-01163-3 (PMC10344787; doi:10.1038/s41556-023-01163-3)

Uncropped full-length pictures of IB membranes

Extended Data Fig 10c. ARHGAP35

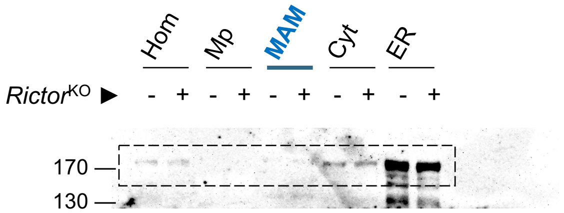

Extended Data Fig 10c. CDC42EP1

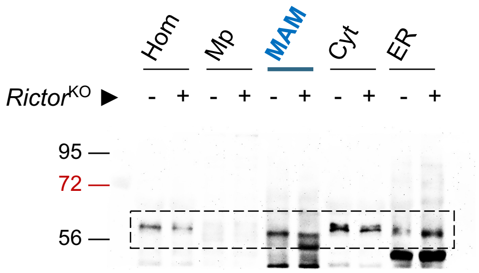

Extended Data Fig 10c. ARHGDIA

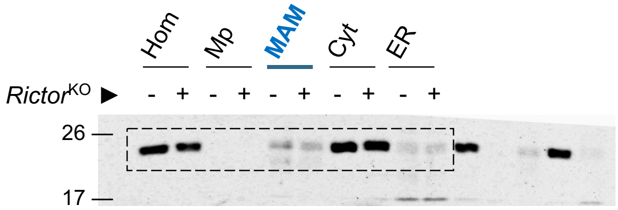

Extended Data Fig 10c. Ponceau

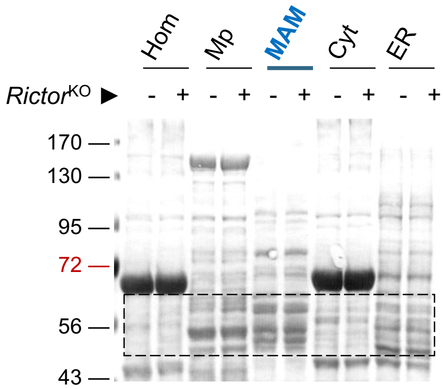

Extended Data Fig 10d. ARHGAP35

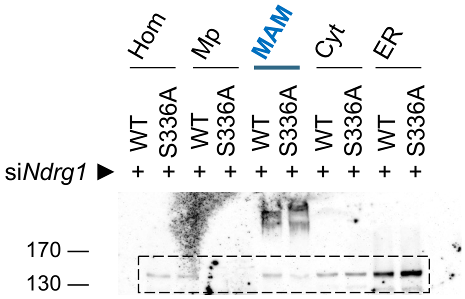

Extended Data Fig 10d. CDC42EP1

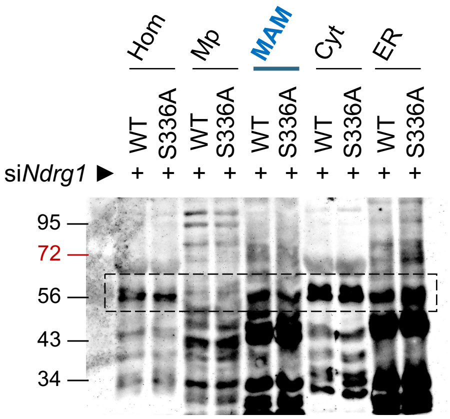

Extended Data Fig 10d. ARHGDIA

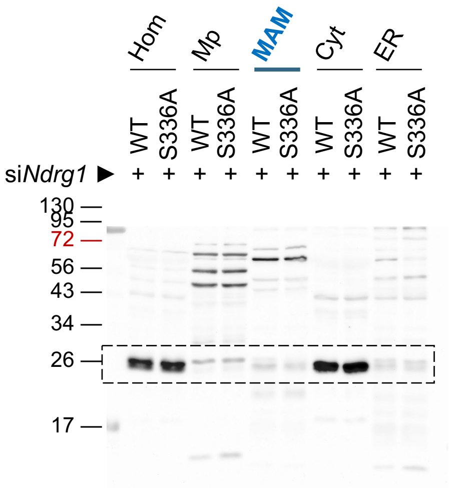

Extended Data Fig 10d. Ponceau

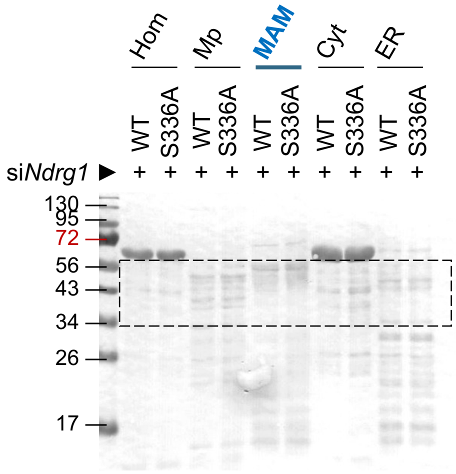

Supplement: Source Data Extended Data Fig. 10 — Unprocessed western blots for Extended Data Fig. 10. [file 41556_2023_1163_MOESM34_ESM.pdf]
